# Volatile Emission of Pear Tree (*Pyrus communis*) and Olfactory Perception of Pear Psyllids (*Cacopsylla pyri* and *C. pyrisuga*) are Affected by Elevated Tropospheric Ozone Concentration

**DOI:** 10.1007/s10886-025-01642-x

**Published:** 2025-08-27

**Authors:** Alicia Koßmann, Jannicke Gallinger, Margit Rid-Moneta, Christine Becker, Annette Reineke, Jürgen Gross

**Affiliations:** 1 Institute for Plant Protection in Fruit Crops and Viticulture, Julius Kühn-Institute, Federal Research Institute for Cultivated Plants, Schwabenheimer Str. 101, D-69221 Dossenheim, Germany; 2 Plant Chemical Ecology, Technical University of Darmstadt, Schnittspahnstr. 4, D-64287 Darmstadt, Germany; 3 Department of Crop Protection, Geisenheim University, Von-Lade-Str. 1, D-65366 Geisenheim, Germany

**Keywords:** Plant-insect-interaction, Climate change, Ozone increase, Olfaction, Plant volatiles, Pear psyllid

## Abstract

Climate change and rising tropospheric ozone concentration are impacting ecosystems in various ways and can influence the volatile-mediated interactions between insects and their host plants. In this study, individuals of the psyllid species *Cacopsylla pyri* and *C. pyrisuga* as well as their host plant *Pyrus communis* were exposed to elevated but naturally occurring ozone concentrations. The effects on the olfactory perception of the insects and the volatile emission of the plants were examined. Ozone exposure affected the olfactory perception of *C. pyri* and *C. pyrisuga.* While the perception threshold of hexanal increased in *C. pyri*, the perception of the tested compounds was not impaired in *C. pyrisuga*. Behavioural tests with ozone exposed *C. pyri* revealed an unchanged repellent effect of nonanal, while hexanal and a synthetic volatile blend became repellent to this species. These findings show only minimal direct effects of moderate ozone concentrations on olfactory perception of pear psyllids. In contrast, chemical analysis of the host plant odours showed a dramatic change in the volatile composition during ozone exposure, with a statistically significant decrease of terpenes and an increase of aldehydes and ketones, showing a greater impact of ozone on the emitter than on the receiver in the pear-psyllid system. This study highlights that moderate ozone levels can alter volatile emissions, potentially disrupting host plant location and impacting other insect–plant interactions that depend specifically on volatile-mediated communication.

**Supplementary Information:**

The online version contains supplementary material available at 10.1007/s10886-025-01642-x.

## Introduction

Increasing ground-level (tropospheric) ozone concentration is a driver of the progressive climate change. This so-called greenhouse gas is formed by photochemical reactions of mainly volatile organic substances (VOCs) such as hydrocarbons, alcohols, aldehydes, and nitrogen oxides (NO_x_), but also carbon monoxide and methane in the troposphere (EPA 2012; WHO 2006). These precursor compounds are mostly of anthropogenic origin (WHO 2008). In general, it can be assumed that the increase of the precursors is correlated to the growth of the human population, economic and technical development and changes in agriculture and climatic conditions (WHO 2008). In Germany, the tropospheric ozone concentrations varied between 180 and 310 µg/m^3^ during the last two decades. It is estimated that ground-level ozone concentrations have doubled to tripled during the last century (Marenco et al. 1994; Volz and Kley 1988).

As a reactive molecule, ozone triggers oxidative stress in plants (Guidi et al. 2009; Kangasjärvi et al. 2005) and insects (Shaghaghian et al. 2014; Sousa et al. 2012) affecting different parts of the ecosystems. Direct harmful effects are reported for several living organisms (Dıaz-de-Quijano et al. 2012; Kampa and Castanas 2008; Krupa et al. 2001). Due to its property as an oxidising agent this gas can cause changes in plant or animal tissue or physiology (Bergweiler and Manning 1999; Black et al. 2007). Further, ozone interacts differentially with several biogenic organic volatile compounds (BVOCs) in the atmosphere (Atkinson and Arey 2003; Calogirou et al. 1999; Holzinger et al. 2005; Yu et al. 1999). Plants produce a variety of VOCs via different metabolic pathways: terpenoids, compounds with aromatic rings, fatty acid derivates, amino acid derivates (Baldwin 2010). In the environment, VOCs form a landscape of scents that mediate inter- and intraspecific interactions. This includes the attraction of pollinators (Raguso 2008), plant-plant communication (Baldwin et al. 2006), above- and below-ground herbivore defence (Ali et al. 2012; Degenhardt et al. 2009; Hiltpold and Turlings 2012; Unsicker et al. 2009), and protection against pathogens (Gross et al. 1998; Huang et al. 2012). Accordingly, such a complex system might be severely disturbed by ozone, either through altered BVOC compositions due to plant stress responses or through the reaction of BVOCs in the gas phase (Agathokleous et al. 2017; Duque et al. 2019; Farré-Armengol et al. 2016; Fuentes et al. 2013; Khaling et al. 2016). Overall, ozonation is a multi-phase process that involves a series of stepwise reactions, including the formation of reactive intermediates and secondary oxidation products. The degradation process is strongly influenced by the type and chemical structure of the compounds. In particular, carbon–carbon double bonds (C = C) are highly susceptible to oxidation, whereas saturated compounds are generally less affected. The interaction between psyllids and their host plants is a complex system which is primarily chemically mediated by scents (Gross 2016; Gross et al. 2022; Mayer et al. 2011) and offers numerous points of attack for ozone. The olfactory perception of the insects can be restricted or the scent bouquets can be modified in such a way that insects are not able to recognise their plant anymore. Not much is yet known about effects of ozone on the olfactory system of insects but some studies indicate a negative impact on the olfactory perception of specific volatiles (Farré-Armengol et al. 2016; Vanderplanck et al. 2021;). While most studies have been conducted with beneficial insects such as pollinators (e.g., Agaonidae, Apidae) (Farré-Armengol et al. 2016; Vanderplanck et al. 2021), it is also important to investigate whether ozone disrupts the host-finding process of phytophagous species that are harmful for agricultural plants, such as psyllids. In contrast to the negative impacts on the olfactory system, degradation products can include compounds that are detectable and behaviourally relevant to insects. For example, highly oxidized compounds such as small carbonyls have been shown to be perceived by drosophilid antennae and can elicit attractive behavioural responses (Venkateswaran et al. 2023).

The aim of this study was to understand how ozone influences the VOC-mediated interaction between pear psyllids *Cacopsylla pyri* or *C. pyrisuga* and their host plant *Pyrus communis*. Pear leaf suckers (Hemiptera: Psyllidae) are important pests on European pear (Civolani 2012). Psyllids do not only extract nutrients from their host plant, they also excrete honeydew (Völkl et al. 1999), which promotes the growth of sooty moulds on leaves and fruits (Le Goff et al. 2019). Furthermore, both species vector the cell wall-lacking phytoplasma *‘Candidatus* Phytoplasma pyri’, which is transmitted through phloem sucking of the psyllids and causes the disease pear decline (PD) which can weaken or even kill trees (Civolani 2012; Jarausch et al. 2019; Lethmayer et al. 2011; Kuĉerová et al. 2007; Seemüller and Schneider 2004). Beside multiple similarities between the two psyllid species, there is one important difference. While *C. pyri* is a polyvoltine psyllid that stays on the pear tree all over the year, the univoltine *C. pyrisuga* has a more complex life cycle. It migrates between the pear tree (reproduction host plant) and evergreen conifers (overwintering host plant) (Jarausch et al. 2019; Lazarev 1975). Psyllid infestations in pear orchards cause significant yield losses especially in Eurasia, so control of pear psyllids is important for farmers. In order to develop biological plant protection strategies, it is important to understand, which chemical cues are responsible for the insects to find their host plant (Gross and Gündermann 2016).

In this study we aimed to investigate the impact of ozone on both parts of insect-plant recognition: the volatile emission of *P. communis* and the volatile perception through *C. pyri* and *C. pyrisuga*. Therefore, the headspace from pear tree was analysed and compared between trees under ambient air conditions and artificially increased ozone concentrations supplied during the sampling. Electroantennography was used to determine possible impacts on the antennal perception by *C. pyri* and *C. pyrisuga.* Furthermore, the olfactory attraction of electroantennographic active compounds was tested with an olfactometer.

## Methods and Materials

### Insects

**Fig. 1:**
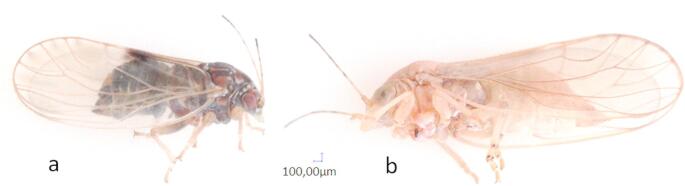
Female psyllid species *Cacopsylla pyri* (summer form) (a) and *Cacopsylla pyrisuga* (emigrant) (b)

Wild psyllids were caught spring (March/April) 2021 from pear trees at the experimental field of the Julius Kühn-Institut (JKI) in Dossenheim, Germany: *Cacopsylla pyrisuga* remigrants and summer form of *Cacopsylla pyri* (Fig. 1a). Both psyllid species were reared in cages (BugDorm, MegaView Science Co, Taiwan, 47.5 × 47.5 × 93 cm) with one potted pear tree (*Pyrus communis* cv. `Williams Christ´ grafted on cv. `Kirchensaller Mostbirne´). Maintenance of the insects was conducted in a climate chamber under the following conditions: 16/8 h day night cycle at 25 °C during photophase and 15 °C during scotophase. *C. pyrisuga* remigrants were removed after they had laid eggs. Experiments were conducted only with the hatched emigrants with a maximum age difference of six weeks caused by the procedure of the experiments (Fig. 1b). *C. pyri* was bred permanently after capture. For this, adults were transferred into new cages after oviposition to obtain insects belonging to the same known age for the experiments.

### Ozone Exposure

Ozone was generated with a stable UVP ozone generator (Analytik Jena, Jena, Germany) and an activated carbon (Sigma Scientific, Micanopy, USA) filtered air stream. Ozone enriched air was directed into the reaction chamber (volatile collection chamber for plants and desiccator for insects) through polytetrafluoroethylene (PTFE) tubing. Ozone concentration in the reaction chamber was monitored and recorded with an ozone sensor (Series 500 Monitor, Aeroqual, Auckland, New Zealand). Pear trees were exposed to ozone in a volatile collection chamber. Ozone was introduced at the upper inlet. The system was operated for 30 min prior the VOC sampling to establish a stable ozone concentration. A concentration of 164 ± 6 µg/m^3^ ozone was hold for 30 min during volatile collection (details see below). For insect exposure, about 20 females per species were put in gauze bags and placed into a 3 L glass desiccator in which the generated ozone was discharged. Psyllids were exposed for 3 hours to 462 ± 8 µg/m^3^ ozone (*C. pyrisuga*) respectively 473 ± 12 µg/m^3^ ozone (*C. pyri*). Subsequent experiments were conducted 90 min after the end of ozone exposure.

### Chemicals

Following chemicals were used for electrophysiological and behavioural tests with insects. Three aldehydes hexanal, heptanal, and nonanal were tested as single compounds. Furthermore, a volatile blend according to Czarnobai de Jorge et al. (2024) consisting of typical pear volatiles (17% cis-3-hexenylacetate, 3.4% methyl salicylate, 3.4% α-pinene, 66.7% ocimene, 3.1% cis-3-hexenol; and as solvent 6.4% methylene chloride (DCM) was examined. Dilutions were made with DCM. All chemicals were purchased from Merck ((Sigma, Sigma Aldrich, Fluka), Darmstadt, Germany).

### Electroantennography (EAG)

EAG was used to assess the impact of ozone exposure on the olfactory sensitivity of insects to plant-emitted VOCs. To determine whether ozone exposure of the insects alters the perception threshold for VOCs dose-response curves were measured for specific compounds. Single female adults of *C.pyrisuga* or *C. pyri* were placed head first in a 200 µl pipette tip (George et al. 2016). The front end of the pipette tip was cut off diagonally so that only the head with the right antenna and the mesonotum of the psyllid was exposed. A small piece of cotton wool behind the insect fixed it in the required position. The pipette tip with the prepared insects was fastened 1 cm in front of an air stream filtered through activated carbon and humidified, with a continuous flow of 1.23 L/min (Stimulus Controller Typ CS-55, SYNTECH, Hilversum, Netherlands). Glass capillaries (0.58 mm I.D., Science Products, Hofheim, Germany) were pulled with a glass Micro Electrode Pipette Puller (PN-3, Narishige, Japan) to make them thinner and sharp. These capillaries were filled with Ringer solution (NaCl 7.5 g, KCl 0.35 g, CaCl2 0.21 g, 1 L H_2_O) and pulled over the electrodes. The indifferent glass electrode was inserted through the mesonotum into the insect. Into the other glass electrode, the last segment of the distal end of the insect antenna was placed. The signal from the electrode was recorded using EAGPro software (Ockenfels Syntech^®^ GmbH, Buchenbach, Germany) and was amplified 10-fold by an IDAC-4 amplifier (Ockenfels Syntech^®^ GmbH, Buchenbach, Germany). Substances were diluted in DCM. For the recordings 1 µg, 10 µg, 100 µg and 1000 µg of the substances were freshly pipetted on a filter paper (Type 413, VWR Collection). DCM was used as negative control. Hexanal (1000 µg) was used as a positive control (Gallinger et al. 2020). The filter paper was inserted into a glass Pasteur pipette (22.86 cm) and solvent was allowed to evaporate for about 3 min. The glass Pasteur pipette was then connected to a stimulus generator (IDAC-2, Ockenfels Syntech^®^ GmbH, Buchenbach, Germany). This device generated an air puff which took the sample volatiles over the antenna with an air flow of 1.46 L/min and a duration of 1 s. A refractory period of at least 3 min between the puffs with the single compounds or blends of compounds allowed the antenna to recover. Furthermore, there was a control puff (clean air) in the middle of the refractory period. EAG recordings always began with an air control, followed by a negative control and a positive control puff. This was repeated at the end of the recording to verify the reactivity of the antenna. The substances were tested with increasing concentrations from 1 µg up to 1000 µg, but the order of the substances was chosen randomly. One individual female psyllid was tested for all different substances and doses in a row. For each substance 10 to 12 replicates were performed with ozone exposed and unexposed *C. pyri* and *C. pyrisuga*. Electroantennographic data for unexposed psyllids and the pear volatile blend were provided by Czarnobai de Jorge et al. (2024).

### Olfactometer Assay

To investigate the impact of ozone-exposure on the attractiveness of the VOCs to psyllids, olfactometer assays were conducted with ozone-exposed and unexposed psyllids. The assays were performed as described in Gallinger et al. (2020) and Czarnobai de Jorge et al. (2022). A dynamic air flow Y-shaped olfactometer (entrance arm length: 12.5 cm, test arm length: 7.5 cm, inner diameter: 1 cm, angle: 75°) was placed on a board in an angle of 45° from the horizontal plane. All experiments were conducted in a dark room between 09:00 and 16:00. A light source (LED-Lupenleuchte, Purelite, UK; 280 lx) was mounted above the middle of the olfactometer. Glass olives with a frit were attached to both ends of the test arms. The olives contained the substance or solvent control pipetted on a filter paper. Filter papers were replaced after one hour. A continuous, activated carbon filtered (ICAF 2 × 6, Sigma Scientific, Micanopy, USA) and humidified airflow of 0.04 L/min was pumped through the olives into the test arms. The flow was adjusted with plastic valves and controlled by a digital flowmeter (MASS-STREAM, M + W Instruments, Allershausen, Germany) before the experiments to ensure an equal airstream in both test arms. The preference of adult *C. pyri* females was tested for 1000 µg of single VOCs (hexanal, heptanal, nonanal) as well as 3 µl of the 1:10 diluted volatile blend. For this, single psyllids were caught with small plastic vials the day before the experiments and stored at 7 °C in the dark to increase their motivation. One hour before the behavioural assay started the insects were kept at room temperature (21–24 °C). Psyllids must walk freely from the plastic vial into the entrance arm. They were observed for 5 min after entering the olfactometer or until deciding between the substance or the solvent control. A decision was recorded when the insects entered at least the half length of the test arm. The entire system was twisted after five replicates to prevent side effects. Psyllids were recorded as “no choice” psyllids if they did not reach half-length of one of the test arms within 5 min. After every behavioural assay, all tubes, valves, and glass olfactometers were cleaned with ethanol (70%) and heated at 220 °C (except plastic valves: 60 °C) for 5 h.

### Headspace Sampling

**Fig. 2:**
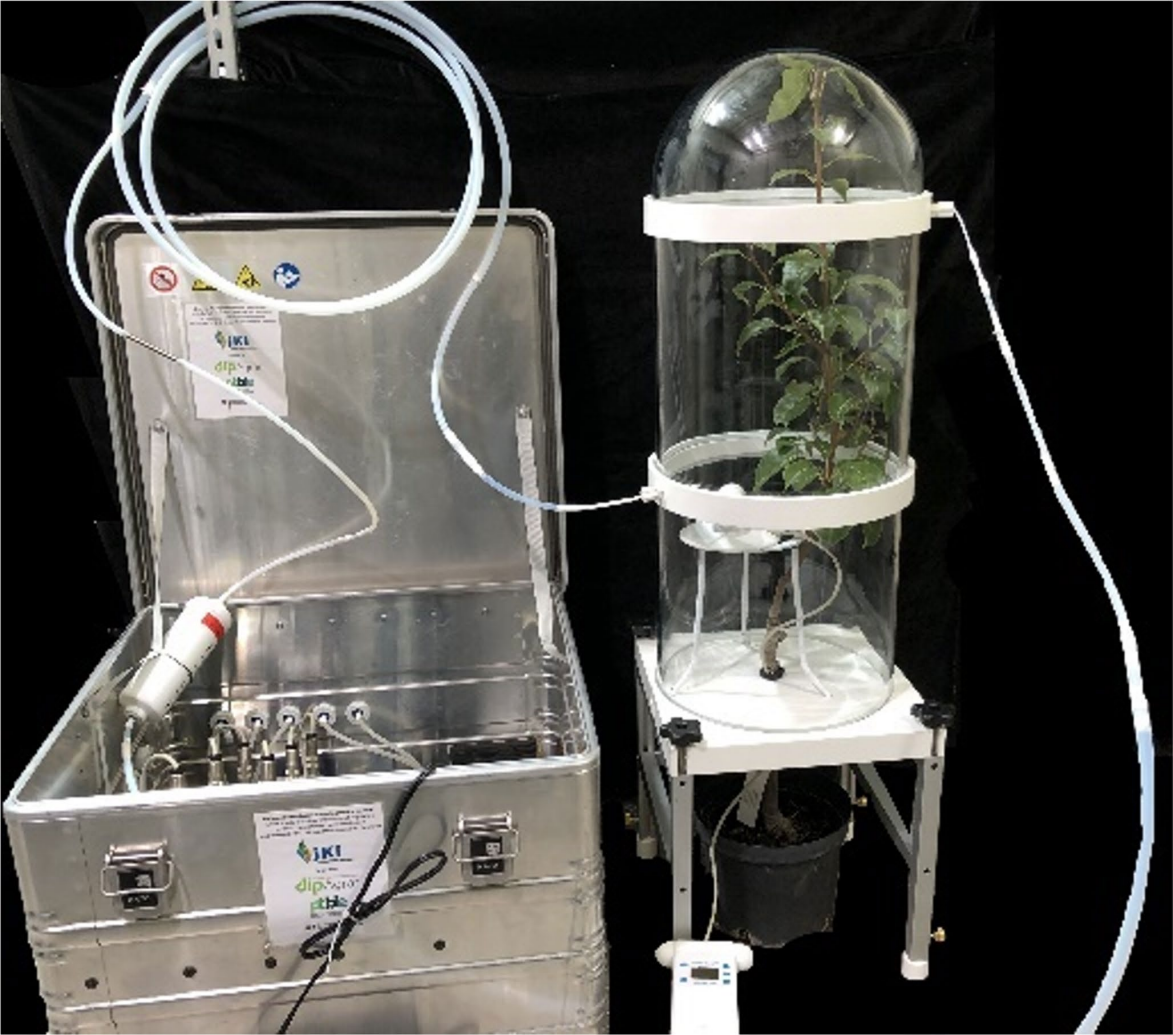
Headspace sampling set up. Pear tree (*Pyrus communis*) placed in a sectional adjustable hight volatile glass collection chamber connected via PTFE tubing to a mobile headspace sampling device. An ozone sensor was placed inside the collection chamber. Filtered air or ozone enriched air was supplied through the upper port, volatiles were collected through the lower port

Headspace samples from eleven pear trees *Pyrus communis* cv. `Williams Christ´ grafted on cv. `Kirchensaller Mostbirne´ were taken before and during ozone exposure using a glass sectional adjustable height volatile collection chamber (Sigma Scientific, Micanopy, USA) under controlled conditions (20 °C, 50% humidity) in a climate chamber (Fig. 2). All trees used for headspace sampling had a similar growth, with no yellow or curled leaves. The whole pear tree except for the lower section of the stem and the roots in the pot were placed in the collection chamber. The stem in the guillotine stand was coated with a charcoal “wrap” for sealing. Between the glass parts were plastic connection rings with a port for Teflon tubes (Fig. 2). The upper port was connected to the ozone setup which supplied charcoal filtered air (ICAF2 × 6, Sigma Scientific, Micanopy, USA) and ozone into the collection chamber. The lower port was connected to a mobile 6-channel headspace sampling device as described by Rid et al. (2016) modified as described in Gross et al. (2019). This consisted of vacuum pumps (KNF Neuberger GmbH, Freiburg, Germany) connected to mass flow controllers (M + W Instruments GmbH, Leonhardsbuch, Germany). For each tree one sample with ambient, charcoal filtered air and one with elevated ozone was taken. Sampling was conducted between 9:00 and 13:00 with a duration of 30 min at an air flow of 1000 mL/min. Volatiles were trapped on stainless steel, prepacked sample tubes with Tenax 35/60 sorbent (Markes, Neu-Isenburg, Germany). After the sampling, the tubes were closed with PTFE-coated brass compression caps (Swagelok, Perkin Elmer). All tubes used for headspace sampling setup consisted of PTFE and were cleaned with ethanol (70%) and heated at 220 °C for 5 h. The volatile collection chamber was cleaned with ethanol (70%) and heated at 80 °C for 1 h followed by 60 °C for 5 h. PTFE parts from the setup were cleaned with ethanol (70%) and heated at 220 °C for 5 h.

### Thermodesorption – GC-MS

Sample analysis was performed with an automated thermal desorber (TurboMatrix™ ATD 650, PerkinElmer, Rodgau, Germany) connected to a gas chromatograph coupled with mass spectrometer (GC–MS). Desorption of sample tubes lasted 10 min at 250 °C. Throughout the tube desorption process the cold trap (Tenax TA) was held at − 20 °C. Then the trap was heated at a rate of 99 K/s to 250 °C and desorbed for 1 min and compounds were separated using a PerkinElmer^®^ Clarus^®^ 680 GC system equipped with a nonpolar Elite-5MS (30 m x 0.25 mm id x 0.25 μm film thickness, Perkin Elmer). As carrier gas helium (Helium 6.0, Linde, Munich, Germany) at a pressure of 130 kPa was used for splitless injection to the coupled quadrupole inert mass selective detector (Perkin Elmer, Rodgau, Germany). GC temperature program started with an initial oven temperature of 40 °C and was held for 1 min. Then the temperature increased linear at a rate of 5 K/min to 180 °C, followed by a rate of 20 K/min to 280 °C. This final temperature was held for 6 min. The ion source temperature was 180 °C. The GC inlet line temperature was 250 °C. The quadrupole mass detector was operated in the electron impact (EI) mode at 70 eV. All data were obtained by collecting the full-scan mass spectra within the range of 35–350 m/z.

### Identification and Quantification with AMDIS

The evaluation was carried out according to the protocol of Gross et al. (2019). For identification and quantification of the volatile compounds, chromatograms were analysed with the “Automated Mass Spectral Deconvolution and Identification System” (AMDIS, V2.73; National Institute of Standards and Technology (NIST), Boulder, USA). Fragmentation patterns (mass spectra) and retention indices (RI) were automatically compared with those of reference substances stored in our own library. Mass spectra with no match were compared with reference spectra of the NIST08 library (National Institute of Standards and Technology, vers. 08 (NIST08); Mass Spectral Search Program (MS-Search). If the spectra matched > 90%, the reference spectrum from NIST was added to the library and the respective RI value was added from the NIST Chemistry WebBook (Nist Mass Spectrometry Data Center; 20.10.2021). When selecting the RI from the database, the technical data such as temperature programme, column type and diameter, gas, layer thickness and the material of the stationary phase of the GC had to match or be comparable. Some of the substances could also be identified by comparing the RT, RI and mass spectrum with standards already measured by the same system (Weintraub and Gross 2013) (Tab. S1, supplementary material). Following criteria were used for identification: match factor had to be ≥ 80%, the relative retention index deviation must be ≤ 5% from reference value. The default settings for deconvolution were: component width: 32; adjacent peak subtraction: one; resolution: low; shape requirements: low; level: very strong; maximum penalty: 50, “no RI in library”: 20 and a signal to noise ration higher than 50. Uncertain identified compounds (match < 80%) were set as “known unknowns” and named after their RI values. Compounds were excluded from further calculations if they occurred in less than 10% of the total sample numbers. The peak areas were integrated and relative proportions were calculated. For this the sum of the selected compounds was set as 100%. The resulting compositional data set was used for statistical analysis.

### Statistics

All statistical analyses were done in R version 4.0.4 (R Core Team 2021) and the packages RVAideMemoire (Hervé 2021) and vegan (Oksanen et al. 2020). Graphs were created using the ggplot2 package (Wickham 2016). To calculate significant differences between EAG signals toward the tested volatiles and the respective air controls a Wilcoxon matched pairs signed-rank test was used. The signal strength toward the volatiles resulted from the response elicited by the compound and the subtraction of the respective air control and was used to compare antennal reactions of unexposed and ozone exposed psyllids using a Mann-Whitney-Test. Preference of unexposed or ozone exposed psyllids for synthetic compounds in the olfactometer assays was evaluated with the binominal test. To compare the volatile pattern of unexposed and ozone exposed pear trees a compositional dataset of the peak area of 61 substances was calculated for each plant sample. As a multivariate test for discrimination of groups a permutational multivariate analysis of variance (PERMANOVA, Anderson (2001) of the compositional datasets was used. Moreover, a permutational analysis of multivariate dispersions (PERMDISP, Anderson (2006) test for homogeneity of multivariate dispersion was carried out. The result of PERMDISP indicates if the dispersion of the volatile pattern and so the significant PERMANOVA is based on location and not on dispersion effects. Both tests were conducted with Bray–Curtis dissimilarities (Brückner and Heethof, 2017). Further, the emission (absolute peak areas) of single compounds from pear trees with and without ozone exposure was compared by Wilcoxon matched pairs signed-rank tests.

## Results

### Volatile Analysis

A total number of 61 compounds was detected in chromatograms of the unexposed and ozone exposed pear trees (Tab. S2, supplementary material).

**Fig. 3:**
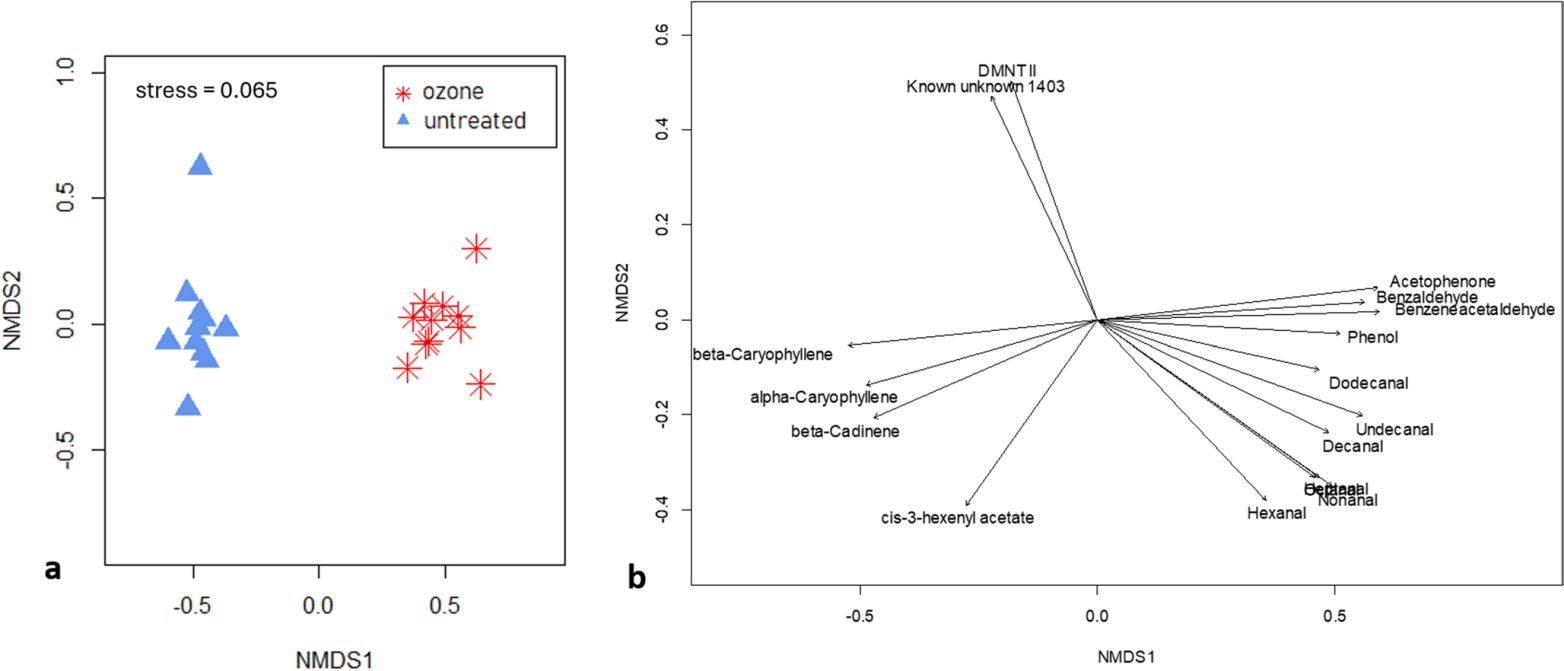
**a** Non-metric multidimensional scaling (NMDS) plot visualizing dissimilarities (Bray-Curtis) of the single volatile profiles (61 substances) of unexposed (blue triangles) and ozone exposed (red stars) pear trees (*n* = 11). **b** Substances responsible for the separation are shown with arrows in different directions

A statistically significant difference between the volatile profiles of the unexposed and ozone-exposed plants is demonstrated (*PERMANOVA*, df = 1, R^2^ = 57.76, *N* = 10.000, *p* < 0.001. The PERMDISP, which followed the PERMANOVA, could not give an indication if the separation is due the dispersion within the two groups or due to different location (*PERMDISP*, df = 1, F = 8.29, *p* = 0.008). The non-metric multidimensional scaling plot shows a clear separation of the unexposed and under ozone collected samples (Fig. 3a).

**Fig. 4:**
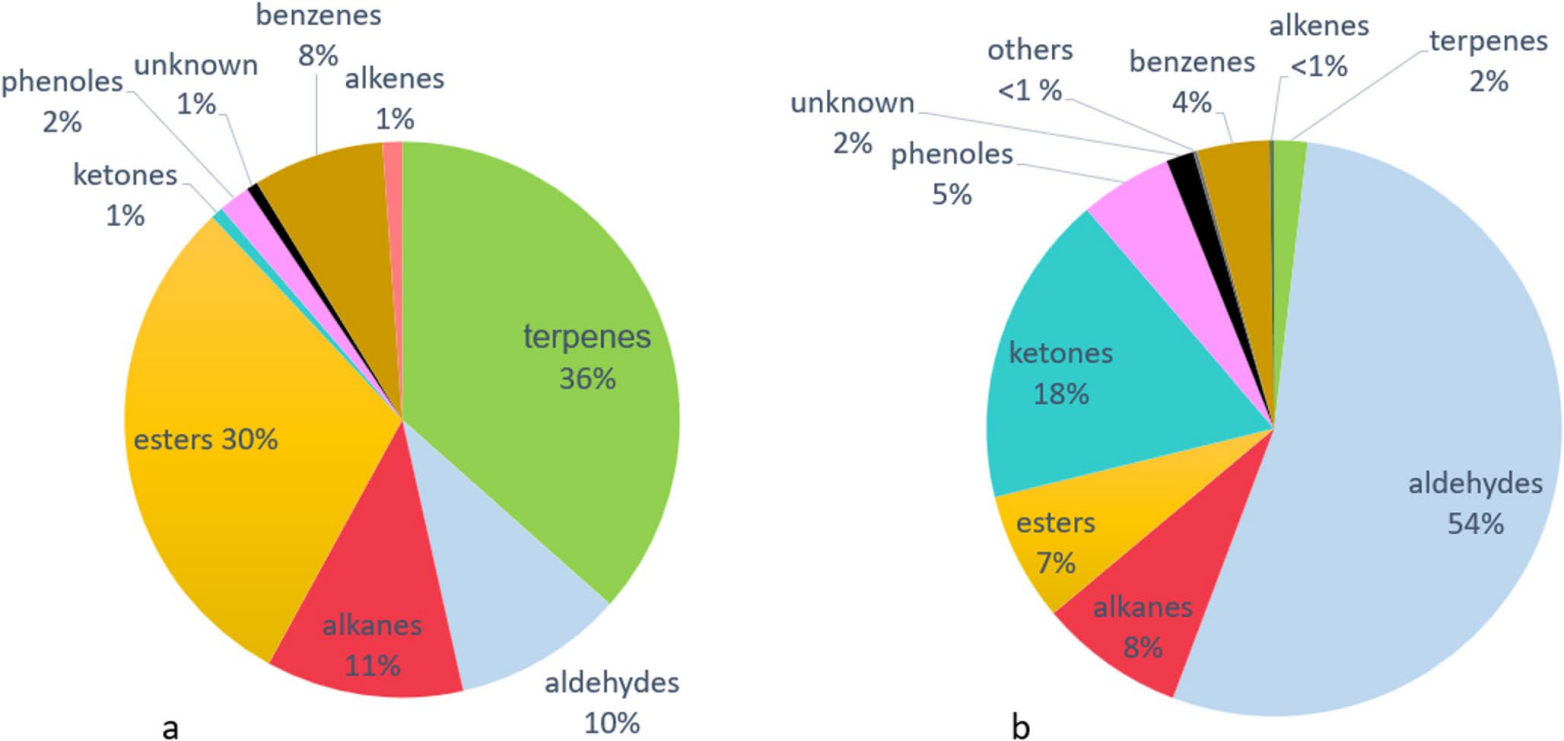
Mean proportion of different substance classes (terpenes, aldehydes, alkanes, ester, ketones, phenols, benzenes, alkenes, other (benzothiazole) and not identified substances (unknown). a VOC spectra of unexposed pear trees. b VOC spectra of ozone exposed (164 ± 6 µg/m3) pear trees (*n* = 11)

The relative proportions of different volatile classes changed under ozone exposure (Fig. 4a, Tab. S2). The headspace of the unexposed trees was dominated by terpenes (36%) and esters (30%). Cis-3-hexenylacetate was the most abundant substance in unexposed volatile samples with an average of 26.6%. During ozone exposure aldehydes had the largest proportion of the volatile bouquet (54% Fig. 4b). Nonanal and benzaldehyde had the largest proportion with 16% each. Terpenes and esters had decreased considerably when VOCs were exposed and accounted for 2% and 7% of the total bouquet. The percentage of ketones changed from 1% for the control plants to 18% for the ozone-exposed pear trees. The relative proportion of benzenes was halved in the headspace of the exposed plants, while that of phenols approximately doubled. The proportion of alkanes remained almost unchanged.

**Fig. 5:**
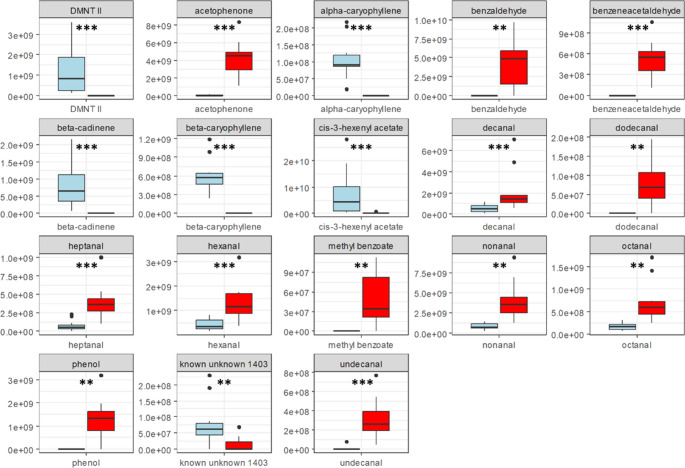
Absolute peak-areas of compounds that are responsible for the separation of the volatile profiles of unexposed (blue, *n* = 11) and ozone treated (red, *n* = 11) pear trees. Significant differences (*p* < 0.01) are marked with asterisks (*Wilcoxon matched pairs signed-rank test*). Asterisks indicate the level of significance of *Wilcoxon matched pairs signed-rank test* (*, *P* < 0.05; **, *P* < 0.01; ***, *P* < 0.001)

Sesquiterpenes including α-caryophyllene, β-caryophyllene, β-cadinene, and the ester cis-3-hexenylacetate were primarily responsible for the separation of the groups in one direction. Separation in the other direction was caused by aldehydes: benzaldehyde, benzene acetaldehyde, dodecanal, undecanal, decanal, heptanal, octanal, nonanal, and hexanal. In addition, acetophenone and phenol contributed to the splitting of the unexposed and ozone exposed samples (Fig. 3b). Absolute peak area values of these substances were significantly different between ozone exposed and unexposed pear trees (Fig. 5).

*Electroantennography. C. pyri: s*ingle compounds emitted from *P. communis* that showed electrophysiological responses in *C. pyri* were selected for the electroantennographic recordings (Koßmann et al., unpublished) to investigate the effect of ozone on the perception of psyllids. The DCM solvent control puff did not elicit a significant antennal reaction.

**Fig. 6:**
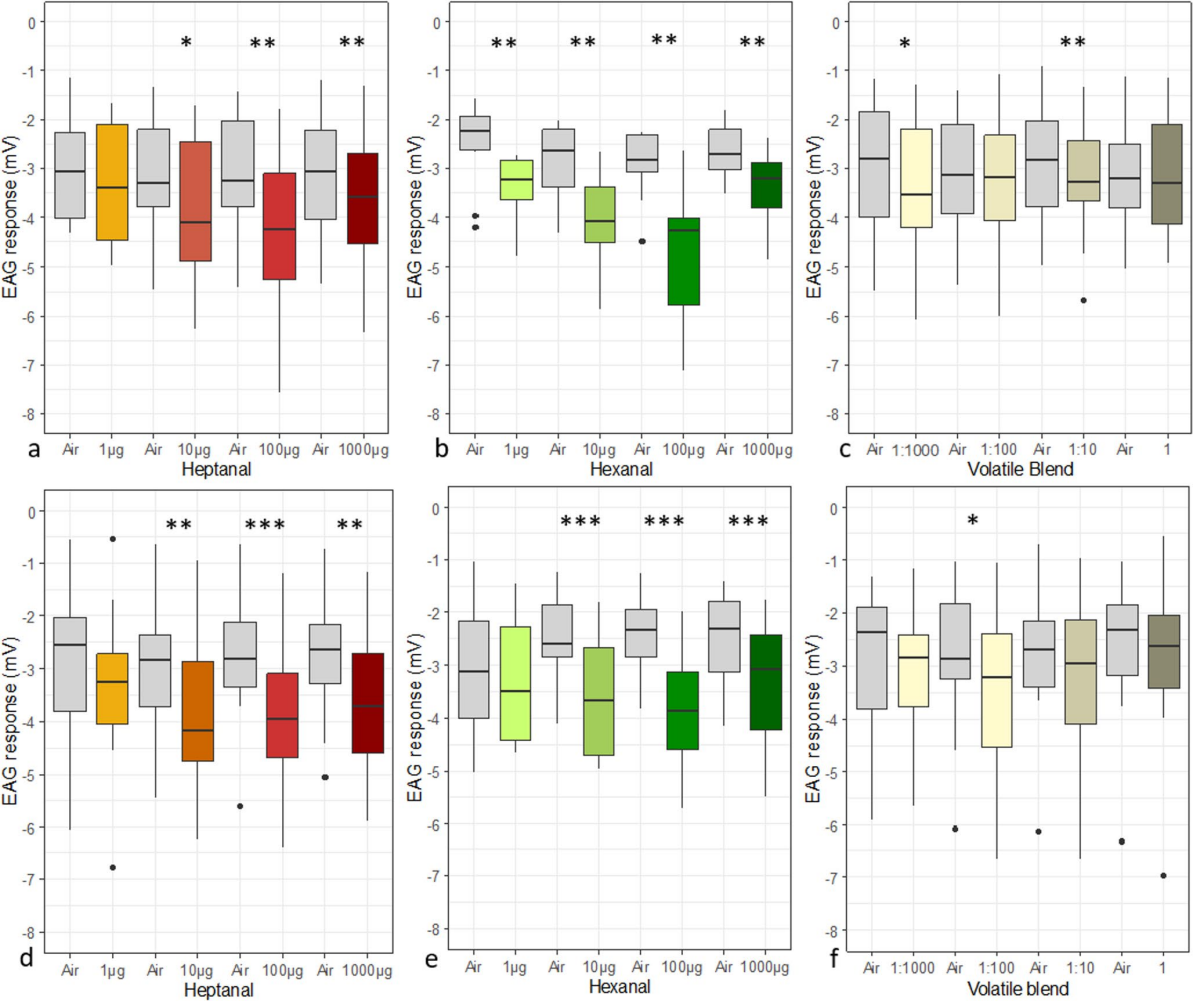
Antennal responses of unexposed (*n* = 10) (a-c) and ozone exposed (473 ± 12 µg/m^3^, *n* = 12) (d-f) female *C. pyri* to puffs with 1 µg, 10 µg, 100 µg, 1000 µg heptanal (a)(d), hexanal (b)(e), different dilutions of the volatile blend 1:1000, 1:100, 1:10, undiluted (data from Czarnobai de Jorge et al. 2024) (c) (f), and air control (grey). Asterisks indicate the level of significance of *Wilcoxon matched pairs signed-rank test* (*, *P* < 0.05; **, *P* < 0.01; ***, *P* < 0.001)

After the exposure of *C. pyri* to increased ozone concentrations the perception threshold of heptanal remained unchanged at 10 ug (*Wilcoxon matched pairs signed-rank test*, unexposed: V = 3, *p* < 0.01, exposed: V = 1, *p* < 0.01) (Fig. 6a, d). For hexanal an increase of the perception threshold from 1 µg measured from unexposed psyllids (*Wilcoxon matched pairs signed-rank test*, V = 1, *p* < 0.01) to 10 µg from ozone exposed psyllids (*Wilcoxon matched pairs signed-rank test*, V = 0, *p* < 0.001) was detected (Fig. 6b, e). For the volatile blend there was a shift from the 1:1000 dilution to the 1:100 diluted mixture (*Wilcoxon matched pairs signed-rank test*, unexposed: V = 6, *p* < 0.05, exposed: V = 9, *p* < 0.05) (Fig. 6c, f).

There were no statistically significant differences between the reaction strengths of ozone exposed and unexposed *C. pyri* to heptanal (Table 1). A statistically significant reduction was observed for 1 µg hexanal after ozone exposure (*Wilcoxon-Mann-Whitney rank sum test*, W = 24, *p* < 0.05) (Table 1). Larger differences occurred with the volatile blend. The maximum reaction strength after ozone exposure was observed for the 1:100 diluted solution. For unexposed psyllids the maximum reaction was for the 1:10 dilution of the volatile blend (Table 1).

**Table 1:** Mean reaction strength (mV) of unexposed female *C. pyri* and *C. pyrisuga* and after a 3-hour Ozone exposure (473 ± 12 µg/m^3^ *C. pyri*; 462 ± 8 µg/m^3^ *C. pyrisuga*). Reaction strength is calculated as the difference between the electrophysiological reaction to the volatile substance and air

	Unexposed C. pyri	Ozone exposed C. pyri	Unexposed C. pyrisuga	Ozone exposed C. pyrisuga
Heptanal				
1 µg	−0.26 ± 0.40	−0.33 ± 0.50	−1.08 ± 0.49	-
10 µg	−0.71 ± 0.63	−0.93 ± 0.88	−1.39 ± 0.69	-
100 µg	−1.16 ± 0.78	−1.10 ± 0.69	−2.13 ± 0.63	-
1000 µg	−0.60 ± 0.43	−0.91 ± 0.76	−2.74 ± 1.34	-
Hexanal				
1 µg	−0.88 ± 0.57	−0.23 ± 0.60 *	−0.41 ± 0.40	−0.74 ± 0.73
10 µg	−1.18 ± 0.64	−1.10 ± 0.74	−0.82 ± 0.29	−1.25 ± 0.54 *
100 µg	−1.81 ± 1.04	−1.46 ± 0.71	−1.32 ± 0.33	−1.74 ± 0.91
1000 µg	−0.77 ± 0.39	−0.83 ± 0.40	−0.80 ± 0.57	−1.48 ± 1.30
Volatile blend^1^				
1:1000	−0.35 ± 0.44	−0.25 ± 0.55	−0.55 ± 1.22	−0.14 ± 0.76
1:100	−0.15 ± 0.26	−0.59 ± 0.81	−0.96 ± 0.77	−0.89 ± 0.68
1:10	−0.39 ± 0.32	−0.47 ± 0.90	−0.49 ± 0.49	−0.71 ± 0.36
1	0.03 ± 0.34	−0.26 ± 0.86	−0.82 ± 1.32	−0.18 ± 0.39

^1^Data from Czarnobai de Jorge et al. 2024

asterisks indicate significant difference between ambient conditions (C. pyri *n* = 10; C. pyrisuga *n* = 10) and ozone exposure (C. pyri *n* = 12; C. pyrisuga *n* = 10) (Mann-Whitney-Test (*, *P* < 0.05))

**Fig. 7:**
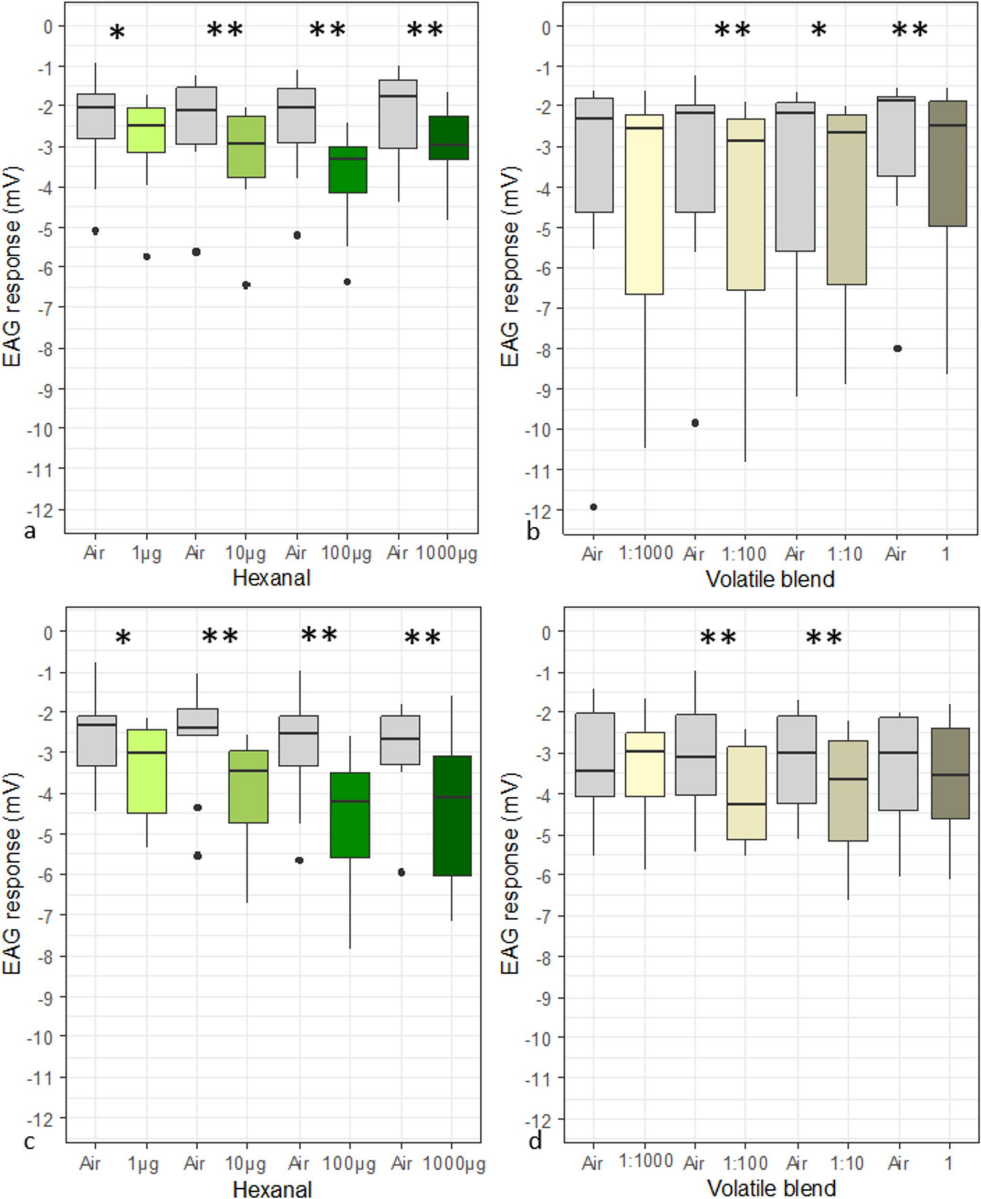
Antennal responses of unexposed (a-b) and ozone exposed (462 ± 8 µg/m^3^) (c-d) (*n* = 10). *C. pyrisuga* to puffs with 1 µg, 10 µg, 100 µg, 1000 µg hexanal (a)(c), different dilutions of the volatile blend 1:1000, 1:100, 1:10, undiluted (data from Czarnobai de Jorge et al. 2024 (b) (d), and air control (grey) (*n* = 10). Asterisks indicate the level of significance of *Wilcoxon matched pairs signed-rank test* (*, *P* < 0.05; **, 0.01)

*Electroantennography C. pyrisuga*: as a single compound hexanal was tested for antennal responses of *C. pyrisuga.* Data for the volatile blend were taken from Czarnobai de Jorge et al. 2024. After ozone exposure there was no shift of the perception threshold (hexanal: *Wilcoxon matched pairs signed-rank test*, V = 4, *p* < 0.05; volatile blend: *Wilcoxon matched pairs signed-rank test*, V = 0, *p* < 0.01). The insects’ perception threshold of hexanal was at 1 µg of the aldehyde (*Wilcoxon matched pairs signed-rank test*, V = 2, *p* < 0.05) (Fig. 7a, c). For the volatile blend, the first significant antennal reaction compared to the respective air control was observed for the 1:100 dilution (*Wilcoxon matched pairs signed-rank test*, V = 0, *p* < 0.01) (Fig. 7d). After exposure the insects did not respond significantly different to the undiluted blend (*Wilcoxon signed-rank test*, V = 15, *p* = 0.23) (Fig. 7b, d).

The reaction strength remained unchanged after ozone exposure compared to the unexposed psyllids, with the exception for 10 µg hexanal (Fig. 7 and Table 1). A puff with 10 µg hexanal elicited a statistically significantly higher reaction strength from exposed females compared to non-exposed individuals (*Wilcoxon Mann-Whitney rank sum test*, W = 79, *p* < 0.05) (Table 1).

### Olfactometer Assay

**Fig. 8:**
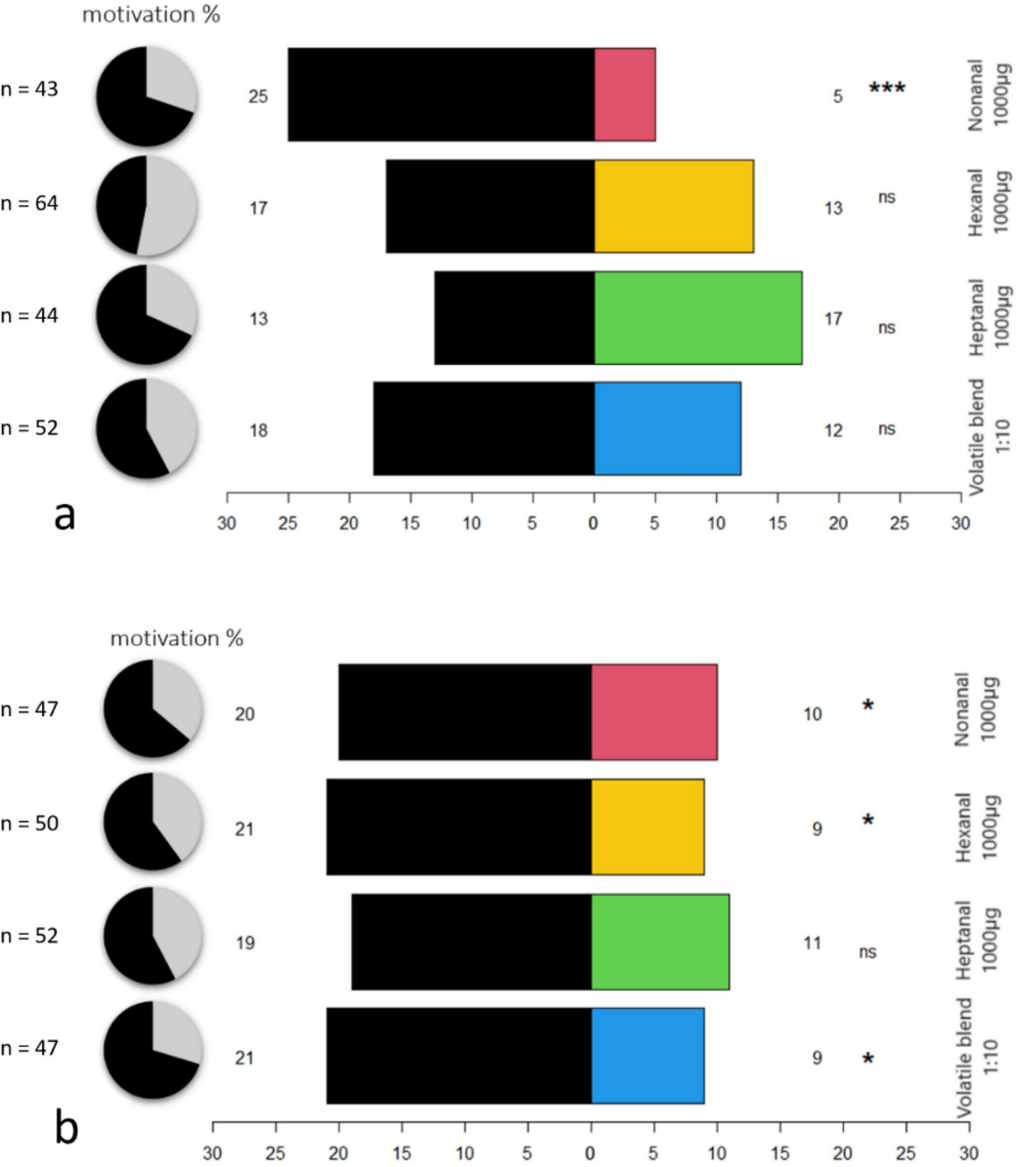
Choice of *C. pyri* females in a Y-Tube olfactometer. Tested substances are presented in colours, solvent control in black. The pie charts on the right present the number of psyllids that made a choice (black) and that did not (light grey) (n.s.= not significant, * = significant *p* < 0.05, *** = high significant *p* < 0.001, *binominal test* **a** unexposed psyllids **b** psyllids after a 3-hour ozone exposure with 461 ± 11 µg/m^3^

Without ozone exposure *C. pyri* females showed an avoidance of 1000 µg nonanal (*binominal test*, *p* < 0.001) (Fig. 8a). Untreated psyllids did not show any preference or avoidance to the volatile blend, heptanal, and hexanal (*binominal test*, *p* > 0.05). During the olfactometer tests with 1000 µg hexanal, the insects exhibited the lowest level of motivation, with only 46.9%. The psyllids demonstrated avoidance behavior by attempting to escape the olfactometer and avoid entering the test arms (personal observation).The highest motivation was for 1000 µg nonanal 69.8%.

After ozone exposure *C. pyri* showed an avoidance of 1000 µg nonanal, hexanal, and the volatile blend (*binomial test*, *p* < 0.05). For heptanal the insects had no preference (Fig. 8b).

## Discussion

The degradation of the terpenes and the increase of the aldehydes were the driving factors in the changes of the volatile profile of the pear trees due to ozone.

An exposure with a naturally occurring ozone concentration of 164 ± 6 µg/m^3^ revealed quantitative and qualitative changes in the volatile profile of pear trees. Absolute values and therefore the proportions of single components changed: while various terpenes and esters decreased, several aldehydes and ketones increased significantly.

Ozone has known direct and indirect effects on plants. Direct effects are photooxidative stress and phytotoxicity caused by this high reactive molecule (Guidi et al. 2009; Kangasjärvi et al. 2005). It can lead to a decreased photosynthetic capacity (Long and Naidu 2002; Loreto et al. 2004) and, in response, a lower carbon availability (Sitch et al. 2007). Intracellularly, ozone leads to the activation of defence genes and biochemical cascades (Baier et al. 2005; Fiscus et al. 2005; Heath 2008; Kangasjärvi et al. 2005). In this context, VOC emission, such as isoprene, can protect the plant from ozone-damage. The gas reacts with the emitted VOCs, resulting in concentrations that are less or non-toxic to plants (Loreto et al. 2001). The identified O_3_-induced volatiles are produced from different biosynthetic pathways (Beauchamp et al. 2005; Heiden et al. 1999). While terpene emission can be altered by the activity of the MVA synthesis pathway, GLV emission can result from activation of the LOX pathway (Feussner and Wasternack 2002). Some of these biosynthetic pathways are also involved in herbivore defence, attraction of predators and parasitoids, or plant-plant communication. One example is the denaturation of cellular lipids (Pell et al. 1997), lipid oxidation and the production of C6 volatiles (Beauchamp et al. 2005). This occurs mainly during insect feeding and causes the release of GLVs. Among these is hexanal, which was significantly elevated by ozone exposure. The ozonolysis (Criegee 1975) of the functional group of the alkenes (C = C double bond) of epicuticular waxes and volatiles in the dermal tissues of the leaves can lead to the production of new VOCs from the carbonyl family, such as ketones or aldehydes (Calogirou et al. 1999; Fruekilde et al. 1998). These findings match the results of this work, where the proportion of aldehydes was increased by 44%, that of ketones by 17%. Increased aldehyde emission was already detected for several plants in context with ozone exposure (Acton et al. 2018; Farré-Armengol et al. 2016; Mofikoya et al. 2018). In contrast, terpenes and certain green leaf volatiles react readily with ozone, leading to the formation of various compounds in the gas and particle phase (Atkinson and Arey 2003; Calogirou et al. 1996, 1999; Holzinger et al. 2005; Yu et al. 1999). Monoterpenes, especially sesquiterpenes, are susceptible to degradation by ozonolysis due to their chemical structure (C = C double bond) (Bonn and Moortgat 2003; Winterhalter et al. 2009). Similarly to our results, the reduction of several terpenes by ozone in the headspace of plants is shown for other plant species (Acton et al. 2018; Farré-Armengol et al. 2016; Li and Blande 2015; Mofikoya et al. 2018; Pinto et al. 2007a, b). Interestingly we observed the formation of aldehydes, but no alkanoic acids were detected, suggesting incomplete oxidation under the given ozone-to-VOC ratio.

The effects of ozone on the olfactory perception of psyllids were species and compound specific. Interestingly, the sensitivity towards hexanal was opposed in the species, increased in *C. pyrisuga*, but impaired for low hexanal concentrations in *C. pyri*.

Current knowledge about the effects of ozone on the insect olfactory system is still limited. Studies on pollinators show that ozone impacted the perception of various volatiles (Dötterl et al. 2016; Vanderplanck et al. 2021). Important factors thereby might be the duration of exposure, the ozone concentration but also the type of receptor. In addition, different insect species may have different sensitivities to ozone (Vanderplanck et al. 2021).

Olfactory perception of hexanal by *C. pyri* was negatively affected by ozone exposure. The perception threshold shifted from 1 µg to 10 µg. Similar effects for a 3-hour long exposure with 400 µg/m^3^ were shown for fig wasps, where a significant reduced perception of 1 µg benzyl alcohol compared to control conditions was measured (Vanderplanck et al. 2021). Significant lower antennal responses to different benzaldehyde and cis-3-hexenylacetate concentrations were also demonstrated for bumblebee (*Bombus terrestris*) (Vanderplanck et al. 2021) and honey bee (*Apis mellifera*) (Dötterl et al. 2016), while the perception of nonanal or 2-phenyl alcohol remained unchanged for these two pollinators despite ozone exposure (Dötterl et al. 2016; Vanderplanck et al. 2021). Additionally, under comparable experimental conditions a 3-hour ozone exposure (420 µg/m^3^) caused a significant decrease in the antenna signals of *Lobesia botrana* in response to the sexual pheromone (Nieszporek, unpublished). Therefore, it is likely that a longer exposure period at moderately high ozone concentrations is necessary to induce negative effects on VOC perception.

A shorter (one-hour) exposure of fig wasps (*Blastophaga psenes*) to 400 µg/m^3^ ozone led to statistically significant stronger antennal responses to benzyl alcohol compared to control in EAG-recordings (Vanderplanck et al. 2021). We achieved similar results in our study with *C. pyrisuga*. Ozone-exposed *C. pyrisuga* females (3 h, 462 µg/m^3^) showed a general higher reaction strength to hexanal than under control conditions, and a statistically significant higher sensitivity to 10 µg of the VOC. However, it must be considered that in between the electroantennographic studies of *C. pyrisuga* with or without ozone exposure was unfortunately a gap of four to six weeks. This univoltine psyllid species has a complex life cycle which includes migration and emigration. As the tested insects were long-living emigrants who need to have a high fitness to migrate to their higher placed overwintering site, a higher age of *C. pyrisuga* must not have to be accompanied with a lower vitality, but perhaps the reverse. The later tested insects might have a higher fitness and therefore lasted longer than the younger individuals at the first experiments. This could be a possible explanation for the stronger antennal responses after ozone exposure.

The results of the electrophysiological experiments in combination with the behavioural assays suggest that ozone has a possible damaging impact on certain odorant-binding proteins or receptors which are responsible for olfactory perception. Investigations with this oxidative gas have already shown that ozone reacts with amino acids and can thus impair the functionality of proteins (Mudd et al. 1969). Different receptor types appear to be damaged by ozone in different ways, which explains the detected compound-specific rather than general effects on psyllid behaviour. It is necessary to identify which receptor types are found in the sensilla of psyllids and whether their protein structure is sensitive to oxidation. Additionally further studies are needed to investigate whether the damage is irreversible or if the damaged structures can regenerate again.

In contrast to the findings of Czarnobai de Jorge et al. (2024), the identical volatile blend did not elicit attraction in our behavioural experiments. This discrepancy may be due to differences in the psyllid origin: while Czarnobai de Jorge et al. (2024) used wild-caught psyllids, the psyllids in our study were reared under controlled climate chamber conditions. The EAG recordings indicated that hexanal tended to be perceived less strongly after ozone exposure of the psyllids. As a result, hexanal may have been perceived as less repellent, and this reduction in sensitivity was likely just sufficient for the insects to enter the olfactometer, although they subsequently avoided the source. Similarly, no increased frequency of psyllids leaving the olfactometer was observed when the 1:10 diluted volatile blend was tested. Our analyses clearly show that VOCs react to varying degrees with ozone. The plant odour bouquet changes qualitatively, as well as quantitatively, which may disrupt insect-host plant communication (Himanen et al. 2009; Li and Blande 2015). To be able to recognise the host plant, often not only the presence or content of a particular VOC is important, but the presence of a specific blend of different VOCs in a certain ratio or relation to each other (Bruce and Pickett 2011; Bruce et al. 2005). It has been shown that exposing flowers to 120 ppb (= 240 µg/m^3^) ozone significantly reduced their attractiveness for *B. terrestris* (Farré-Armengol et al. 2016). Pinto et al. (2007c) did not find negative effects of ozone on the orientation and behaviour of predatory mites and parasitic wasps in tritrophic systems beside a degradation of several herbivore induced plant volatiles. Highlighting that the impact of ozone on insect plant interactions is depending on the atmospheric stability of the VOCs used for the communication in the specific system more atmospherically stable volatile compounds may be used for communication in some insect plant interactions systems.

In pear psyllids it is not yet known which particular cues are essential for host plant recognition. For *C. picta*, a closely related psyllid species, β-caryophyllene plays a key role in the host plant detection (Mayer et al. 2008a, b). Therefore, the great impact of ozone on the terpene emission of plants could be of high relevance. If the insects use chemically stable compounds or additionally other signals like colour or mechanosensory cues, ozone may not have such a significant effect on host attraction. It was recently shown that *C. pyri* was attracted by green colour with wavelengths ranging from 525 to 537 nm both in lab and field studies (Czarnobai de Jorge et al. 2023).

The results of the electrophysiological and behavioural tests along with the increased amount of both aldehydes under ozone, suggest that the pear psyllids may have difficulties in recognising their host plants. The impact of ozone might be more affecting migrating species such as *C. pyrisuga*, because, after completing their migration from overwintering on conifers, they must locate suitable host plants for reproduction (Jarausch et al. 2019). Difficulties in host plant identification during this critical phase could significantly impact their survival and reproductive success.

Dual-choice tests with pear trees under ozone and unexposed trees or an air control could clarify whether the psyllids recognise and visit their food source or have a preference. Also, long term ozone exposure of plant and insects could be interesting. Further it has to be clarified how long the impact of ozone lasts. It is unlikely that *C. pyri* will unlikely give up its food source due to a short-term change of the volatile profile.

Furthermore, climate change affects not only the concentration of tropospheric ozone, but also other factors like the concentration of CO_2_ in the atmosphere, and causes elevated temperatures. These factors may interact together with tropospheric ozone. It was shown recently that the relative release of single compounds by pear trees changed in response to CO_2_ increase in the field (Gallinger et al. 2023). But differences in VOC release were inconsistent over time and between study years, indicating interactions with other climate parameters, such as ozone or temperature. Even though insect-plant interaction can rely on specific volatile compounds and specific mixtures of compounds, respectively, the changes of VOC patterns in this field study did not impact the host choice behaviour of *C. pyri* females (Gallinger et al. 2023). Thus, the interactions between different factors affected by climate change should be in the focus of future research.

Beyond its relevance for chemical communication between species, further knowledge is essential for developing resilient semiochemical-based management strategies and ensuring their effectiveness under projected environmental conditions. Future research should focus on guaranteeing the robustness of artificially dispersed semiochemicals against ozone and other factors by identifying stable compounds and dispersal methods, as well as testing their performance in both current and future field scenarios.

Overall, the results of this study highlight how ozone can interfere with plant–insect interactions, primarily through alterations in the volatile profile of pear trees, but also by affecting insect olfactory perception. Nonetheless, a certain degree of plasticity is essential for insects to cope with changing environmental conditions. Therefore, the extent to which these relatively small and specific changes in antennal perception impair host-finding behaviour in pear psyllids remains to be clarified and needs further investigation.

## Supplementary Information

Below is the link to the electronic supplementary material.

Supplementary Material 1

Supplementary Material 2

Supplementary Material 3

## Data Availability

Data is provided within the manuscript or supplementary information files.

## References

[CR1] Acton WJF, Jud W, Ghirardo A, Wohlfahrt G, Hewitt CN, Taylor JE, Hansel A (2018) The effect of ozone fumigation on the biogenic volatile organic compounds (BVOCs) emitted from *Brassica napus* above- and below-ground. PLoS One 13:e0208825. 10.1371/journal.pone.0208825 30532234 10.1371/journal.pone.0208825 PMC6287848

[CR2] Agathokleous E, Sakikawa T, Abu ElEla SA, Mochizuki T, Nakamura M, Watanabe M, Kawamura K, Koike T (2017) Ozone alters the feeding behavior of the leaf beetle *Agelastica coerulea* (Coleoptera: Chrysomelidae) into leaves of Japanese white Birch (*Betula platyphylla* var. japonica). Environ Sci Pollut Res 24:17577–17583. 10.1007/s11356-017-9369-7 10.1007/s11356-017-9369-7 28597386

[CR3] Ali JG, Alborn HT, Campos-Herrera R, Kaplan F, Duncan LW, Rodriguez-Saona C, Koppenhöfer AM, Stelinski LL (2012) Subterranean, herbivore-induced plant volatile increases biological control activity of multiple beneficial nematode species in distinct habitats. PLoS One 7:e38146. 10.1371/journal.pone.0038146 22761668 10.1371/journal.pone.0038146 PMC3384653

[CR4] Anderson MJ (2001) A new method for non-parametric multivariate analysis of variance. Austral Ecol 26:32–46. 10.1111/j.1442-9993.2001.01070.pp.x

[CR5] Anderson MJ (2006) Distance-based tests for homogeneity of multivariate dispersions. Biometrics 62:245–253. 10.1111/j.1541-0420.2005.00440.x 16542252 10.1111/j.1541-0420.2005.00440.x

[CR6] Atkinson R, Arey J (2003) Gas-phase tropospheric chemistry of biogenic volatile organic compounds: a review. Atmos Environ 37:197–219. 10.1016/S1352-2310(03)00391-1

[CR7] Baier M, Kandlbinder A, Golldack D, Dietz K-J (2005) Oxidative stress and ozone: perception, signalling and response. Plant Cell Environ 28:1012–1020. 10.1111/j.1365-3040.2005.01326.x

[CR8] Baldwin IT (2010) Plant volatiles. Curr Biol 20:392–397. 10.1016/j.cub.2010.02.052 10.1016/j.cub.2010.02.052 20462477

[CR9] Baldwin IT, Halitschke R, Paschold A, von Dahl CC, Preston CA (2006) Volatile signaling in plant-plant interactions: talking trees in the genomics era. Science 311:812–815. 10.1126/science.1118446 16469918 10.1126/science.1118446

[CR10] Beauchamp J, Wisthaler A, Hansel A, Kleist E, Miebach M, Niinemets Ü, Schurr U, Wildt J (2005) Ozone induced emissions of biogenic VOC from tobacco: relationships between Ozone uptake and emission of LOX products. Plant Cell Environ 28:1334–1343. 10.1111/j.1365-3040.2005.01383.x

[CR11] Bergweiler CJ, Manning WJ (1999) Inhibition of flowering and reproductive success in spreading dogbane (*Apocynum androsaemifolium*) by exposure to ambient ozone. Environ Pollut 105:333–339 15093075 10.1016/s0269-7491(99)00044-5

[CR12] Black VJ, Stewart CA, Roberts JA, Black CR (2007) Ozone affects gas exchange, growth and reproductive development in *Brassica Campestris* (Wisconsin fast plants). New Phytol 176:150–163 17803646 10.1111/j.1469-8137.2007.02163.x

[CR13] Bonn B, Moortgat GK (2003) Sesquiterpene ozonolysis: origin of atmospheric new particle formation from biogenic hydrocarbons. Geophys Res Lett 30:1585. 10.1029/2003GL017000

[CR14] Brückner A, Heethoff M (2017) A chemo-ecologists’ practical guide to compositional data analysis. Chemoecology 27:33–46. 10.1007/s00049-016-0227-8

[CR15] Bruce TJA, Pickett JA (2011) Perception of plant volatiles blends by herbivorous insects – finding the right mix. Phytochem 72:1605–1611. 10.1016/j.phytochem.2011.04.011 10.1016/j.phytochem.2011.04.011 21596403

[CR16] Bruce TJA, Wadhams LJ, Woodcock CM (2005) Insect host location: a volatile situation. Trends Plant Sci 10:269–274. 10.1016/j.tplants.2005.04.003 15949760 10.1016/j.tplants.2005.04.003

[CR17] Calogirou A, Larsen BR, Brussol C, Duane M, Kotzias D (1996) Decomposition of terpenes by ozone during sampling on tenax. Anal Chem 68:1499–1506. 10.1021/ac950803i 21619114 10.1021/ac950803i

[CR18] Calogirou A, Larsen BR, Kotzias D (1999) Gas-phase terpene oxidation products: a review. Atmos Environ 33:1423–1439. 10.1016/S1352-2310(98)00277-5

[CR19] Civolani S (2012) The past and present of pear protection against the pear psylla, *Cacopsylla pyri* L. In: Perveen F(ed) Insecticides—pest engineering. InTech, Rijeka, 385–408. 10.5772/28460

[CR20] Criegee R (1975) Mechanism of ozonolysis. Angew Chem Int Ed Engl 14:745–752. 10.1002/anie.197507451

[CR21] Czarnobai de Jorge B, Hummel HE, Gross J (2022) Repellent activity of clove essential oil volatiles and development of nanofiber-based dispensers against pear psyllids (Hemiptera: Psyllidae). Insects 13:743. 10.3390/insects13080743 36005368 10.3390/insects13080743 PMC9409830

[CR22] Czarnobai de Jorge B, Koßmann A, Hummel HE, Gross J (2024) Evaluation of a push-and-pull strategy using volatiles of host and non-host plants for non-host plants for the management of Pear psyllids in organic farming. Front Plant Sci 15:1375495. 10.3389/fpls.2024.1375495 38841281 10.3389/fpls.2024.1375495 PMC11150531

[CR23] Czarnobai de Jorge B, Meyhöfer R, Jürgens A, Gross J (2023) Preference of pear psyllid (*Cacopsylla pyri*) for specific colour inspires new application in plant protection. J Appl Entomol 147:976–989

[CR24] Dıaz-de-Quijano M, Schaub M, Bassin S, Volk M, Penuelas J (2012) Ozone visible symptoms and reduced root biomass in the subalpine species *Pinus uncinata* after two years of free-air ozone fumigation. Environ Pollut 169:250–257 22410242 10.1016/j.envpol.2012.02.011

[CR25] Degenhardt J, Hiltpold I, Köllner TG, Frey M, Gierl A, Gershenzon J, Hibbard BE, Ellersieck MR, Turlings TCJ (2009) Restoring a maize root signal that attracts insect-killing nematodes to control a major pest. PNAS USA 106:13213–13218. 10.1073/pnas.0906365106 19666594 10.1073/pnas.0906365106 PMC2726344

[CR26] Dötterl S, Vater M, Rupp T, Held A (2016) Ozone differentially affects perception of plant volatiles in Western honey bees. J Chem Ecol 42:486–489. 10.1007/s10886-016-0717-8 27344162 10.1007/s10886-016-0717-8 PMC4947477

[CR27] Duque L, Poelman EH, Steffan-Dewenter I (2019) Plant-mediated effects of ozone on herbivores depend on exposure duration and temperature. Sci Rep 9:19891–19911. 10.1038/s41598-019-56234-z 31882632 10.1038/s41598-019-56234-z PMC6934497

[CR28] EPA (2012) Integrated science assessment of Ozone and related photochemical oxidants (second external review draft). U.S. Environmental Protection Agency, Washington, DC, USA

[CR29] Farré-Armengol G, Peñuelas J, Li T, Yli-Pirilä P, Filella I, Llusia J, Blande JD (2016) Ozone degrades floral scent and reduces pollinator attraction to flowers. New Phytol 209:152–160. 10.1111/nph.13620 26346807 10.1111/nph.13620

[CR30] Feussner I, Wasternack C (2002) The lipoxygenase pathway. Annu Rev Plant Biol 53:275–297. 10.1146/annurev.arplant.53.100301.135248 12221977 10.1146/annurev.arplant.53.100301.135248

[CR31] Fiscus EL, Booker FL, Burkey KO (2005) Crop responses to ozone: uptake, modes of action, carbon assimilation and partitioning. Plant Cell Environ 28:997–1011. 10.1111/j.1365-3040.2005.01349.x

[CR32] Fruekilde P, Hjorth J, Jensen NR, Kotzias D, Larsen B (1998) Ozonolysis at vegetation surfaces: a source of acetone, 4-oxopentanal, 6-methyl-5-hepten-2-one, and geranyl acetone in the troposphere. Atmos Environ 32:1893–1902. 10.1016/S1352-2310(97)00485-8

[CR33] Fuentes JD, Roulston TH, Zenker J (2013) Ozone impedes the ability of a herbivore to find its host. Environ Res Lett 8:014048. 10.1088/1748-9326/8/1/014048

[CR34] Gallinger J, Jarausch B, Jarausch W, Gross J (2020) Host plant preferences and detection of host plant volatiles of the migrating psyllid species *Cacopsylla pruni*, the vector of European stone fruit yellows. J Pest Sci 93:461–475. 10.1007/s10340-019-01135-3

[CR35] Gallinger J, Rid-Moneta M, Becker C, Reineke A, Gross J (2023) Altered volatile emission of pear trees under elevated atmospheric CO₂ levels has no relevance to pear psyllid host choice. Environ Sci Pollut Res 30:43740–43751. 10.1007/s11356-023-25260-w 10.1007/s11356-023-25260-w PMC10076355 36658318

[CR36] George J, Robbins PS, Alessandro RT, Stelinski LL, Lapointe SL (2016) Formic and acetic acids in degradation products of plant volatiles elicit olfactory and behavioral responses from an insect vector. Chem Senses 41:325–338. 10.1093/chemse/bjw005 26857741 10.1093/chemse/bjw005

[CR37] Gross J (2016) Chemical communication between phytopathogens, their host plants and vector insects and eavesdropping by natural enemies. Front Ecol Evol 4:104. 10.3389/fevo.2016.00104

[CR38] Gross J, Gallinger J, Görg L (2022) Interactions between phloem-restricted bacterial plant pathogens, their vector insects, host plants, and natural enemies, mediated by primary and secondary plant metabolites. Entomol Gen 42(2):185–215. 10.1127/entomologia/2021/1254

[CR39] Gross J, Gallinger J, Rid M (2019) Collection, identification, and statistical analysis of volatile organic compound patterns emitted by Phytoplasma infected plants. In: Musetti R, Pagliari L (eds) Phytoplasmas. Springer, New York, pp 333–343. 10.1007/978-1-4939-8837-2_25 10.1007/978-1-4939-8837-2_25 30362015

[CR40] Gross J, Gündermann G (2016) Principles of IPM in cultivated crops and implementation of innovative strategies for sustainable plant protection. In: Horowitz, RA and Ishaaya, I (eds) Advances in Insect Control and Resistance Management, pp 9–26 Springer Science + Business Media B.V., Dordrecht, The Netherlands

[CR41] Gross J, Müller C, Vilcinskas A, Hilker M (1998) Antimicrobial activity of the exocrine glandular secretions, hemolymph and larval regurgitate of the mustard leaf beetle *Phaedon cochleariae*. J Invertebr Pathol 72:296–303 9784354 10.1006/jipa.1998.4781

[CR42] Guidi L, Degl’Innocenti E, Martinelli F, Piras M (2009) Ozone effects on carbon metabolism in sensitive and insensitive *Phaseolus* cultivars. Environ Exp Bot 66:117–125. 10.1016/j.envexpbot.2008.12.005

[CR43] Heath RL (2008) Modification of the biochemical pathways of plants induced by ozone: what are the varied routs to change? Environ Pollut 155:453–463. 10.1016/j.envpol.2008.03.010 18456378 10.1016/j.envpol.2008.03.010

[CR44] Heiden AC, Hoffmann T, Kahl J, Kley D, Klockow D, Langebartels C, Mehlhorn H, Sandermann H, Schraudner M, Schuh G, Wildt J (1999) Emission of volatile organic compounds from ozone exposed plants. Ecol Appl 9:1160–1167. 10.1890/1051-0761(1999)009[1160:EOVOCF]2.0.CO;2

[CR45] Hervé M (2021) RVAideMemoire: Testing and Plotting Procedures for Biostatistics. R package version 0.9–80. https://CRAN.R-project.org/package=RVAideMemoire

[CR46] Hiltpold I, Turlings TCJ (2012) Manipulation of chemically mediated interactions in agricultural soils to enhance the control of crop pests and to improve crop yield. J Chem Ecol 38:641–650. 10.1007/s10886-012-0131-9 22592335 10.1007/s10886-012-0131-9

[CR47] Himanen SJ, Nerg A-M, Nissinen A, Pinto DM, Stewart CN, Poppy GM, Holopainen JK (2009) Effects of elevated carbon dioxide and ozone on volatile terpenoid emissions and multitrophic communication of transgenic insecticidal oilseed rape (*Brassica napus*). New Phytol 181:174–186. 10.1111/j.1469-8137.2008.02646.x 19076723 10.1111/j.1469-8137.2008.02646.x

[CR48] Holzinger R, Lee A, Paw KT, Goldstein UAH (2005) Observations of oxidation products above a forest imply biogenic emissions of very reactive compounds. Atmos Chem Phys 5:67–75. 10.5194/acp-5-67-2005

[CR49] Huang M, Sanchez-Moreiras AM, Abel C, Sohrabi R, Lee S, Gershenzon J, Tholl D (2012) The major volatile organic compound emitted from *Arabidopsis thaliana* flowers, the sesquiterpene (E)-β-caryophyllene, is a defense against a bacterial pathogen. New Phytol 193:997–1008. 10.1111/j.1469-8137.2011.04001.x 22187939 10.1111/j.1469-8137.2011.04001.x

[CR50] Jarausch B, Tedeschi R, Sauvion N, Gross J, Jarausch W (2019) Psyllid vectors. In: Bertaccini A, Weintraub PG, Rao GP, Mori N (eds) Phytoplasmas: plant pathogenic bacteria -II: transmission and management of Phytoplasma –associated diseases. Springer, pp 53–78

[CR51] Kampa M, Castanas E (2008) Human health effects of air pollution. Environ Pollut 151:362–367 17646040 10.1016/j.envpol.2007.06.012

[CR52] Kangasjärvi J, Jaspers P, Kollist H (2005) Signalling and cell death in ozone-exposed plants. Plant Cell Environ 28:1021–1036. 10.1111/j.1365-3040.2005.01325.x

[CR53] Khaling E, Li T, Holopainen JK, Blande JD (2016) Elevated ozone modulates herbivore-induced volatile emissions of *Brassica Nigra* and alters a tritrophic interaction. J Chem Ecol 42:368–381. 10.1007/s10886-016-0697-8 27167383 10.1007/s10886-016-0697-8

[CR54] Krupa S, McGrath MT, Andersen CP, Booker FL, Krupa S, Burkey KO, Chappelka AH, Chevone BI, Pell EJ, Zilinskas BA (2001) Ambient ozone and plant health. Plant Dis J 85:4–12 10.1094/PDIS.2001.85.1.4 30832068

[CR55] Kuĉerová J, Talácko L, Lauterer P, Navrátil M, Fialová R (2007) Molecular tests to determine ‘*Candidatus* Phytoplasma pyri’ presence in psyllid vectors from a Pear tree orchard in the Czech Republic – a preliminary report. Bull Insectol 60:191–192

[CR56] Lazarev MA (1975) New data on the biology of the large Pear psyllid *Psylla Pyrisuga* forst. (Homoptera, Psylloidea) in the Crimea. Entomologicheskoe Obozrenie 54:758–759

[CR57] Le Goff GJ, Lebbe O, Lohaus G, Richels A, Jacquet N, Byttebier V, Hance T (2019) What are the nutritional needs of the pear psylla *Cacopsylla pyri*? Arthropod-Plant Interact 13:431–439. 10.1007/s11829-018-9644-7

[CR58] Lethmayer C, Hausdorf H, Suarez-Mahecha B, Reisenzein H (2011) The importance of psyllids (Hemiptera: Psyllidae) as vectors of phytoplasmas in pome and stone fruit trees in Austria. Bull Insectol 64:255–256

[CR59] Li T, Blande JD (2015) Associational susceptibility in broccoli: mediated by plant volatiles, impeded by ozone. Glob Change Biol 21:1993–2004. 10.1111/gcb.12835 10.1111/gcb.12835 25504925

[CR60] Long SP, Naidu SL (2002) Effects of oxidants at the biochemical, cell, and physiological levels, with particular reference to Ozone. In: Bell JNB, Treshow M (eds) Air pollution and plant life. Wiley, London, pp 69–88

[CR61] Loreto F, Mannozzi M, Maris C, Nascetti P, Ferranti F, Pasqualini S (2001) Ozone quenching properties of isoprene and its antioxidant role in leaves. Plant Physiol 126:993–1000. 10.1104/pp.126.3.993 11457950 10.1104/pp.126.3.993 PMC116456

[CR62] Loreto F, Pinelli P, Manes F, Kollist H (2004) Impact of ozone on monoterpene emissions and evidence for an isoprene-like antioxidant action of monoterpenes emitted by *Quercus ilex* leaves. Tree Physiol 24(4):361–367. 10.1093/treephys/24.4.361 14757575 10.1093/treephys/24.4.361

[CR63] Marenco A, Gouget H, Nédélec P, Pagés JP, Karcher F (1994) Evidence of a long-term increase in tropospheric ozone from Pic du Midi data series: consequences: positive radiative forcing. J Geophys Res 99:16617–16632. 10.1029/94JD00021

[CR64] Mayer CJ, Vilcinskas A, Gross J (2008a) Pathogen-induced release of plant allomone manipulates vector insect behavior. J Chem Ecol 34:1518–1522. 10.1007/s10886-008-9564-6 19031034 10.1007/s10886-008-9564-6

[CR65] Mayer CJ, Vilcinskas A, Gross J (2008b) Phytopathogen lures its insect vector by altering host plant odor. J Chem Ecol 34:1045–1049. 10.1007/s10886-008-9516-1 18600377 10.1007/s10886-008-9516-1

[CR66] Mayer CJ, Vilcinskas A, Gross J (2011) Chemically mediated multitrophic interactions in a plant-insect vector-phytoplasma system compared with a partially nonvector species. Agr Forest Entomol 13:25–35. 10.1111/j.1461-9563.2010.00495.x

[CR67] Mofikoya AO, Kivimäenpää M, Blande JD, Holopainen JK (2018) Ozone disrupts adsorption of *Rhododendron tomentosum* volatiles to neighbouring plant surfaces, but does not disturb herbivore repellency. Environ Pollut 240:775–780. 10.1016/j.envpol.2018.05.031 29778813 10.1016/j.envpol.2018.05.031

[CR68] Mudd JB, Leavitt R, Ongun A, McManus TT (1969) Reaction of ozone with amino acids and proteins. Atmos Environ 3:669–681. 10.1016/0004-6981(69)90024-9 5382204 10.1016/0004-6981(69)90024-9

[CR69] NIST Mass Spectrometry Data Center, Wallace WE director, Retention Indices In: NIST Chemistry WebBook, NIST Standard Reference Database Number 69, Eds. P.J. Linstrom and W.G. Mallard, National Institute of Standards and Technology, Gaithersburg MD, 20899. 10.18434/T4D303 (Stand: 20.10.2021)

[CR70] Oksanen J, Blanchet FG, Friendly M, Kindt R, Legendre P, McGlinn D, Minchin PR, O’Hra RB, Simpson GL, Solymos P, Stevens MHH, Szoecs E, Wagner H (2020) Vegan: community ecology package. R package version 2.5-7. https://CRAN.R-project.org/package=vegan

[CR71] Pell EJ, Schlagnhaufer CD, Arteca RN (1997) Ozone-induced oxidative stress: mechanisms of action and reaction. Physiol Plant 100:264–273. 10.1111/j.1399-3054.1997.tb04782.x

[CR72] Pinto DM, Nerg A-M, Holopainen JK (2007a) The role of ozone reactive compounds, terpenes and green leaf volatiles (GLVs), in the orientation of *Cotesia plutellae*. J Chem Ecol 33:2218–2228. 10.1007/s10886-007-9376-0 17968627 10.1007/s10886-007-9376-0

[CR73] Pinto DM, Tiiva P, Miettinen P, Joutsensaari J, Kokkola H, Nerg A-M, Laaksonen A, Holopainen JK (2007b) The effects of increasing atmospheric ozone on biogenic monoterpene profiles and the formation of secondary aerosols. Atmos Environ 41:4877–4887. 10.1016/j.atmosenv.2007.02.006

[CR74] Pinto DM, Blande JD, Nykänen R, Dong W-X, Nerg A-M, Holopainen JK (2007c) Ozone degrades common herbivore-induced plant volatiles: does this affect herbivore prey location by predators and parasitoids? J Chem Ecol 33:683–694. 10.1007/s10886-007-9255-8 17333375 10.1007/s10886-007-9255-8

[CR75] Raguso RA (2008) Wake up and smell the roses: the ecology and evolution of floral scent. Annu Rev Ecol Evol Syst 39:549–569. 10.1146/annurev.ecolsys.38.091206.095601

[CR76] R Core Team (2021) R: A language and environment for statistical computing. R Foundation for Statistical Computing, Wien, Österreich. URL https://www.R-project.org/

[CR77] Rid M, Mesca C, Ayasse M, Gross J (2016) Apple proliferation Phytoplasma influences the pattern of plant volatiles emitted depending on pathogen virulence. Front Ecol Evol 3:152. 10.3389/fevo.2015.00152

[CR78] Seemüller E, Schneider B (2004) *Candidatus* Phytoplasma mali’, *‘candidatus* Phytoplasma pyri’ and *‘candidatus* Phytoplasma prunorum’, the causal agents of Apple proliferation, Pear decline and European stone fruit yellows, respectively. Int J Syst Evol Microbiol 54:1217–1226. 10.1099/ijs.0.02823-0 15280295 10.1099/ijs.0.02823-0

[CR79] Shaghaghian S, Niakousari M, Javadian S (2014) Application of ozone post-harvest treatment on Kabkab date fruits: effect on mortality rate of Indian meal moth and nutrition components. Ozone Sci Eng 36:269–275. 10.1080/01919512.2013.872555

[CR80] Sitch S, Cox PM, Collins WJ, Huntingford C (2007) Indirect radiative forcing of climate change through ozone effects on the land–carbon sink. Nature 448:791–794. 10.1038/nature06059 17653194 10.1038/nature06059

[CR81] Sousa AH, Faroni LRA, Silva GN, Guedes RNC (2012) Ozone toxicity and walking response of populations of *Sitophilus zeamais* (Coleoptera: Curculionidae). J Econ Entomol 105:2187–2195. 10.1603/EC12218 23356086 10.1603/ec12218

[CR82] Unsicker SB, Kunert G, Gershenzon J (2009) Protective perfumes: the role of vegetative volatiles in plant defense against herbivores. Curr Opin Plant Biol 12:479–485. 10.1016/j.pbi.2009.04.001 19467919 10.1016/j.pbi.2009.04.001

[CR83] Vanderplanck M, Lapeyre B, Brondani M, Opsommer M, Dufay M, Hossaert-McKey M, Proffit M (2021) Ozone pollution alters olfaction and behavior of pollinators. Antioxidants 10:636. 10.3390/antiox10050636 33919364 10.3390/antiox10050636 PMC8143334

[CR84] Venkateswaran V, Alali I, Unni AP, Weißflog J, Halitschke R, Hansson BS, Knaden M (2023) Carbonyl products of ozone oxidation of volatile organic compounds can modulate olfactory choice behavior in insects. Environ Pollut 337:122542. 10.1016/j.envpol.2023.122542 37717892 10.1016/j.envpol.2023.122542

[CR85] Völkl W, Woodring J, Fischer M, Lorenz MW, Hoffmann KH (1999) Ant-aphid mutualisms: the impact of honeydew production and honeydew sugar composition on ant preferences. Oecologia 118:483–491. 10.1007/s004420050751 28307416 10.1007/s004420050751

[CR86] Volz A, Kley D (1988) Evaluation of the montsouris series of ozone measurements made in the nineteenth century. Nature 332:240–242. 10.1038/332240a0

[CR87] Weintraub PG, Gross J (2013) Capturing insect vectors of phytoplasmas. In: Dickinson MJ, Hodgetts J (eds) Phytoplasma: methods and protocols. Springer, New York, pp 61–72. 10.1007/978-1-62703-089-2_6 10.1007/978-1-62703-089-2_6 22987406

[CR88] WHO (2006) Particulate matter, ozone, nitrogen dioxide and sulfur dioxide. Air quality guidelines: global update 2005. WHO Regional Office for Europe, Kopenhagen, Denmark

[CR89] WHO (2008) Health risks of Ozone from long-range transboundary air pollution. World Health Organization Regional Office for Europe, Kopenhagen, Denmark

[CR90] Wickham H (2016) ggplot2: elegant graphics for data analysis. Springer-, New York

[CR91] Winterhalter R, Herrmann F, Kanawati B, Nguyen TL, Peeters J, Vereecken L, Moortgat GK (2009) The gas-phase ozonolysis of beta-caryophyllene (C15H24). Part I: an experimental study. Phys Chem Chem Phys 11:4152–4172. 10.1039/b817824k 19458818 10.1039/b817824k

[CR92] Yu J, Cocker DR, Griffin RJ, Flagan RC, Seinfeld JH (1999) Gas-phase ozone oxidation of monoterpenes: gaseous and particulate products. J Atmos Chem 34:207–258. 10.1023/A:1006254930583

