# Supplementary material for: Volatile Emission of Pear Tree (Pyrus communis) and Olfactory Perception of Pear Psyllids (Cacopsylla pyri and C. pyrisuga) are Affected by Elevated Tropospheric Ozone Concentration
Source: J Chem Ecol. 2025 Aug 27;51(5):86. doi: 10.1007/s10886-025-01642-x (PMC12390884; doi:10.1007/s10886-025-01642-x)
Supplement: Supplementary file 1 — Supplementary Material 1 [file 10886_2025_1642_MOESM1_ESM.docx]

Table S1: Overview of all volatiles identified in headspace samples of pear tree *Pyrus communis* with and without ozone exposure with retention index (RI) and way of identification. Components were identified by comparing mass spectra and retention indices of authentic standard substances purchased from Sigma Aldrich/Merck (SA), Darmstadt, Germany) or because of mass spectra (MS; match > 90 %)

| Volatile organic compounds | RI | origin |  | Volatile organic compound | RI | origin |
| --- | --- | --- | --- | --- | --- | --- |
| Hexanal | 802 | SA |  | RI=1133,6; 12,9036 min | 1133,6 | MS |
| Essigsäurebutylester | 815 | SA |  | Campher | 1145 | SA |
| 2-Hexenal | 855 | SA |  | Sabina Ketone | 1160 | MS |
| Nonan | 900 | SA |  | RI=1160,1; 13,7049 min | 1160,1 | MS |
| Heptanal | 901 | SA |  | Ethylbenzoat | 1171 | SA |
| Cumol | 914 | SA |  | Methylsalicylat | 1195 | SA |
| α-Pinen | 932 | SA |  | n-Dodecan | 1200 | SA |
| Benzaldehyd | 961 | SA |  | Decanal | 1208 | SA |
| β-Pinen | 972 | SA |  | RI=1209,6; 15,1875 min (2,6-dimethyl Undecane) | 1209 | MS |
| 6-Methyl-5-hepten-2-on | 985 | SA |  | Benzothiazol | 1224 | SA |
| Phenol | 989 | MS |  | Ethylsalicylate | 1265 | SA |
| Pseudocumol | 991 | SA |  | RI=1271,0; 16,9695 min | 1271 | MS |
| Decan | 1000 | SA |  | Tridecan | 1300 | SA |
| Octanal | 1004 | SA |  | Undecanal | 1310 | SA |
| cis-3-Hexenylacetat | 1007 | SA |  | α-Cubeben | 1350 | MS |
| Hexylacetat | 1012 | SA |  | α-Copaen | 1374 | MS |
| β-Cymen | 1023 | MS |  | RI=1381,3; 19,9964 min (Sesquiterpen) | 1381,3 | MS |
| Limonen | 1030 | SA |  | 1-Tetradecen | 1391 | SA |
| beta/trans-Ocimen ^a^ | 1040 | SA |  | Tetradecan | 1400 | SA |
| Benzeneacetaldehyd | 1043 | SA |  | RI=1403,7; 20,5972 min (Sesquiterpen) | 1403 | MS |
| Acetophenon | 1065 | SA |  | Dodecanal | 1406 | SA |
| RI=1065,4; 10,8334 min | 1065 | MS |  | β-Caryophyllen | 1419 | SA |
| Linalooloxid (furanoid) | 1090 | SA |  | α-Caryophyllen | 1455 | SA |
| 4-Nonenal | 1094 | MS |  | RI=1464,8; 22,7271 min | 1464 | MS |
| Methylbenzoat | 1095 | SA |  | RI=1485,7; 22,7363 min | 1485 | MS |
| Linalool | 1099 | SA |  | RI=1499,9; 22,9166 min (Sesquiterpen) | 1499 | MS |
| Undecan | 1100 | SA |  | Pentadecan | 1500 | SA |
| Nonanal | 1105 | SA |  | α-Farnesen ^a^ | 1508 | SA |
| 4,8-dimethyl-1(E)3,7-nonatriene (DMNT) | 1115 | SA |  | Butylhydroxytoluol | 1514 | MS |
| allo-Ocimen | 1128 | MS |  | Octylether | 1644 | MS |
| β-Cadinen | 1520 | MS |  |  |  |  |
| ^a^ Identification by a mixure of isomers as a standard substance | | | | | | |
