# Supplementary material for: Volatile Emission of Pear Tree (Pyrus communis) and Olfactory Perception of Pear Psyllids (Cacopsylla pyri and C. pyrisuga) are Affected by Elevated Tropospheric Ozone Concentration
Source: J Chem Ecol. 2025 Aug 27;51(5):86. doi: 10.1007/s10886-025-01642-x (PMC12390884; doi:10.1007/s10886-025-01642-x)
Supplement: Supplementary file 2 — Supplementary Material 2 [file 10886_2025_1642_MOESM2_ESM.docx]

| Tab. S2: Heat map of the proportion of detected volatiles in untreated and ozone exposed pear trees. Low contributing compounds are visualized in red and high contributing compounds in green | | | | | |
| --- | --- | --- | --- | --- | --- |
|  | **untreated** | **ozone** |  | **untreated** | **ozone** |
| **terpenes** |  |  | **esters** |  |  |
| α-caryophyllene | 0.54 | 0.00 | ethyl benzoate | 0.00 | 0.25 |
| α-copaene | 4.03 | 0.33 | cis-3-hexenyl acetate | 26.59 | 0.40 |
| α-cubebene | 0.09 | 0.00 | acetic acid butylester | 1.05 | 0.29 |
| α-farnesene | 0.77 | 0.00 | ethyl salicylate | 0.00 | 0.21 |
| α-pinene | 2.43 | 0.64 | hexylacetate | 0.18 | 0.15 |
| β-cadinene | 3.60 | 0.00 | methyl benzoate | 0.00 | 0.19 |
| β-caryophyllene | 3.08 | 0.00 | methyl salicylate | 2.19 | 5.75 |
| β-cymene | 0.68 | 0.25 | **phenols** |  |  |
| β-cinene | 0.92 | 0.22 | phenol | 0.00 | 5.04 |
| beta/trans-ocimene | 10.50 | 0.03 | butylated hydroxytoluene | 1.56 | 0.02 |
| limonene | 2.83 | 0.05 | **ketons** |  |  |
| linalool | 0.60 | 0.00 | acetophenone | 0.09 | 17.25 |
| unknown sesquiterpene (RI: 1381.3; 19.9964 min) | 0.09 | 0.00 | sabina ketone | 0.56 | 0.27 |
| unknown sesquiterpene (RI: 1403,7; 20,5972 min) | 0.39 | 0.04 | 6-methyl-5-heptene-2-on | 0.07 | 0.02 |
| unknown sesquiterpene (RI:1499.9; 22.9166 min) | 0.36 | 0.00 | **ether** |  |  |
| camphor | 0.12 | 0.01 | octylether | 0.00 | 0.02 |
| allo-ocimene | 0.18 | 0.00 | **alken** |  |  |
| 4,8-dimethyl-1,3,7-nonatrien (DMNT) | 5.24 | 0.00 | 1-tetradecene | 1.13 | 0.27 |
| linalool oxid (furanoid) | 0.10 | 0.30 |  |  |  |
| **aldehydes** |  |  | **benzenes** |  |  |
| decanal | 2.41 | 7.56 | pseudocumol | 7.63 | 4.05 |
| dodecanal | 0.00 | 0.26 | cumol | 0.28 | 0.13 |
| heptanal | 0.31 | 1.58 | **others** |  |  |
| hexanal | 2.19 | 5.63 | benzothiazole | 0.00 | 0.22 |
| nonanal | 3.90 | 16.17 | **unknown** |  |  |
| octanal | 0.90 | 2.88 | RI:1065.4; 10.8334 min | 0.01 | 0.16 |
| undecanal | 0.02 | 1.19 | RI:1160,1; 13.7049 min | 0.07 | 0.00 |
| benzaldehyde | 0.00 | 16.31 | RI:1209.6; 15.1875 min | 0.42 | 0.19 |
| benzeneacetaldehyde | 0.00 | 2.12 | RI:1271.0;16.9695 min | 0.00 | 0.09 |
| 4-nonenal | 0.00 | 0.08 | RI:1464.8; 22.7271 min | 0.50 | 1.29 |
| 2-hexenal | 0.18 | 0.07 | RI:1485.7; 22.7363 min | 0.08 | 0.00 |
| **alkanes** |  |  | 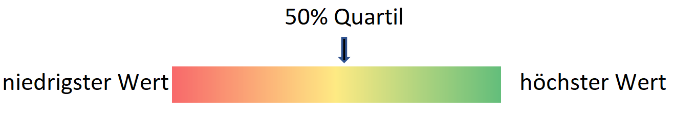 low  high | | |
| decan | 1.89 | 1.40 |  |  |  |
| hexadecan | 0.06 | 0.05 |  |  |  |
| n-dodecan | 1.35 | 1.00 |  |  |  |
| nonan | 0.66 | 0.49 |  |  |  |
| pentadecan | 0.88 | 0.27 |  |  |  |
| tetradecan | 0.48 | 0.52 |  |  |  |
| tridecan | 0.94 | 0.85 |  |  |  |
| undecan | 4.84 | 3.42 |  |  |  |
