# Supplementary material for: Volatile Emission of Pear Tree (Pyrus communis) and Olfactory Perception of Pear Psyllids (Cacopsylla pyri and C. pyrisuga) are Affected by Elevated Tropospheric Ozone Concentration
Source: J Chem Ecol. 2025 Aug 27;51(5):86. doi: 10.1007/s10886-025-01642-x (PMC12390884; doi:10.1007/s10886-025-01642-x)
Supplement: Supplementary file 3 — Supplementary Material 3 [file 10886_2025_1642_MOESM3_ESM.docx]

| Tab. S2: Heat map of the proportion of detected volatiles in untreated and ozone exposed pear trees. Low contributing compounds are visualized in red and high contributing compounds in green | | | | | |
| --- | --- | --- | --- | --- | --- |
|  | **untreated** | **ozone** |  | **untreated** | **ozone** |
| **terpenes** |  |  | **esters** |  |  |
| α-caryophyllene | 0.54 ± 0.32 | 0 | ethyl benzoate | 0 | 0.25 ± 0.55 |
| α-copaene | 4.03 ± 3.05 | 0.33 ± 0.41 | cis-3-hexenyl acetate | 26.59 ± 23.00 | 0.40 ± 0.44 |
| α-cubebene | 0.09 ± 0.07 | 0 | acetic acid butylester | 1.05 ± 0.80 | 0.29 ± 0.26 |
| α-farnesene | 0.77 ± 1.44 | 0 | ethyl salicylate | 0 | 0.21 ± 0.29 |
| α-pinene | 2.43 ± 1.79 | 0.64 ± 0.42 | hexylacetate | 0.18 ± 0.26 | 0.15 ± 0.10 |
| β-cadinene | 3.60 ± 2.17 | 0 | methyl benzoate | 0 | 0.19 ± 0.13 |
| β-caryophyllene | 3.08 ± 1.63 | 0 | methyl salicylate | 2.19 ± 3.31 | 5.75 ± 7.53 |
| β-cymene | 0.68 ± 0.62 | 0.25 ± 0.16 | **phenols** |  |  |
| β-pinene | 0.92 ± 1.23 | 0.22 ± 0.33 | phenol | 0 | 5.04 ± 2.71 |
| beta/trans-ocimene | 10.50 ± 13.49 | 0.03 0.07 | butylated hydroxytoluene | 1.56 ± 1.27 | 0.02 ± 0.02 |
| limonene | 2.83 ± 1.75 | 0.05 ± 0.08 | **ketons** |  |  |
| linalool | 0.60 ± 1.06 | 0 | acetophenone | 0.09 ± 0.12 | 17.25 ± 5.45 |
| unknown sesquiterpene (RI: 1381.3; 19.9964 min) | 0.09 ± 0.09 | 0 | sabina ketone | 0.56 ± 0.86 | 0.27 ± 0.51 |
| unknown sesquiterpene (RI: 1403,7; 20,5972 min) | 0.39 ± 0.32 | 0.04 ± 0.07 | 6-methyl-5-heptene-2-on | 0.07 ± 0.08 | 0.02 ± 0.05 |
| unknown sesquiterpene (RI:1499.9; 22.9166 min) | 0.36 ± 0.29 | 0 | **ether** |  |  |
| camphor | 0.12 ± 0.18 | 0.01 ± 0.02 | octylether | 0 | 0.02 ± 0.03 |
| allo-ocimene | 0.18 ± 0.19 | 0 | **alken** |  |  |
| 4,8-dimethyl-1,3,7-nonatrien (DMNT) | 5.24 ± 5.87 | 0 | 1-tetradecene | 1.13 ± 0.86 | 0.27 ± 0.14 |
| linalool oxid (furanoid) | 0.10 ± 0.18 | 0.30 ± 0.31 |  |  |  |
| **aldehydes** |  |  | **benzenes** |  |  |
| decanal | 2.41 ± 1.39 | 7.56 ± 3.03 | pseudocumol | 7.63 ± 15.89 | 4.05 ± 10.18 |
| dodecanal | 0 | 0.26 ± 0.16 | cumol | 0.28 ± 0.63 | 0.13 ± 0.35 |
| heptanal | 0.31 ± 0.26 | 1.58 ± 0.54 | **others** |  |  |
| hexanal | 2.19 ± 1.50 | 5.63 ± 2.09 | benzothiazole | 0 | 0.22 ± 0.22 |
| nonanal | 3.90 ± 1.75 | 16.17 ± 5.38 | **unknown** |  |  |
| octanal | 0.90 ± 0.51 | 2.88 ± 1.11 | RI:1065.4; 10.8334 min | 0.01 ± 0.02 | 0.16 ± 0.24 |
| undecanal | 0.02 ± 0.08 | 1.19 ± 0.39 | RI:1160,1; 13.7049 min | 0.07 ± 0.13 | 0 |
| benzaldehyde | 0 | 16.31 ± 7.53 | RI:1209.6; 15.1875 min | 0.42 ± 0.26 | 0.19 ± 0.18 |
| benzeneacetaldehyde | 0 | 2.12 ± 0.63 | RI:1271.0;16.9695 min | 0 | 0.09 ± 0.17 |
| 4-nonenal | 0 | 0.08 ± 0.11 | RI:1464.8; 22.7271 min | 0.50 ± 0.44 | 1.29 ± 0.56 |
| 2-hexenal | 0.18 ± 0.43 | 0.07 ± 0.20 | RI:1485.7; 22.7363 min | 0.08 ± 0.11 | 0 |
| **alkanes** |  |  | 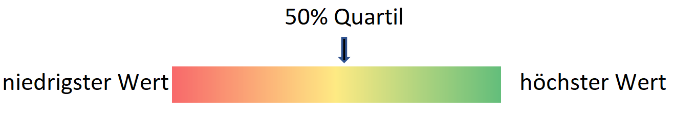 low  high | | |
| decan | 1.89 ± 1.10 | 1.40 ± 0.62 |  |  |  |
| hexadecan | 0.06 ± 0.21 | 0.05 ± 0.15 |  |  |  |
| n-dodecan | 1.35 ± 0.78 | 1.00 ± 0.29 |  |  |  |
| nonan | 0.66 ± 0.45 | 0.49 ± 0.12 |  |  |  |
| pentadecan | 0.88 ± 0.95 | 0.27 ± 0.22 |  |  |  |
| tetradecan | 0.48 ± 0.42 | 0.52 ± 0.29 |  |  |  |
| tridecan | 0.94 ± 0.59 | 0.85 ± 0.49 |  |  |  |
| undecan | 4.84 ± 3.04 | 3.42 ± 2.12 |  |  |  |
